# Formulation of a dual drug-loaded nanoparticulate co-delivery hydrogel system and its validation in rheumatoid arthritis animal model

**DOI:** 10.1080/10717544.2023.2184307

**Published:** 2023-02-28

**Authors:** Prakash Haloi, B. Siva Lokesh, Saurabh Chawla, V. Badireenath Konkimalla

**Affiliations:** a School of Biological Sciences, National Institute of Science Education and Research, HBNI, Bhubaneswar, India; b Training School Complex, Homi Bhabha National Institute, Mumbai, India

**Keywords:** Phenethyl isothiocyanate, rheumatoid arthritis, methotrexate, intra-articular, adjuvant induced arthritis

## Abstract

Rheumatoid arthritis (RA), a systemic autoimmune disease that dramatically affects patients’ quality of life. Given the intricacy of RA’s pathophysiology, no single treatment can completely halt the disease progression. Here, we attempted to treat RA holistically and synergistically by co-delivering methotrexate (MTX), a standard slow-acting anti-rheumatic drug, and phenethyl isothiocyanate (PEITC), a bioactive phytochemical, using a sodium alginate (SA)-pluronic F127 (PF-127) *in situ* hydrogel formulation. Therefore, in the current study, the co-delivery of MTX and PEITC in the nanoparticulate form could help enhance stability and solubility and facilitate greater penetration in the target arthritic tissues. The fabricated MTX NP and PEITC NE were found to have a minimum particle size, PDI, and good zeta potential. Results from *in vitro* release studies showed that MTX and PEITC were simultaneously released from the DD NP HG matrix over 6–7 days through diffusion and erosion mechanisms. An intra-articular (IA) injection of DD NP HG dramatically reduced chronic inflammation in adjuvant-induced arthritis (AIA) rats, delayed the onset of bone erosion, significantly reduced synovitis, and down-regulated the inflammatory cytokine expression. Most notably, the co-delivery strategy almost entirely restored the morphological features of the ankle joints of RA rats. The hepatic and renal function tests indicated good biological safety for DD NP HG in RA conditions. Taken together, these findings indicated that DD NP HG could achieve good anti-inflammatory activity and reverse cartilage disruption through a synergistic effect between two nanoparticulate forms of MTX and PEITC, which can effectively improve the drawbacks of their free forms.

## Highlights

A nanostructured dual-drug loaded smart hydrogel (DD NP HG) was successfully constructed for the intra-articular delivery of MTX and PEITC to the affected joints of RA.

The fabrication of the nanoformulation of both MTX (MTX NP) and PEITC (PEITC NE) aided in mitigating the drawbacks and drug-related side effects of the free form of drugs.

DD NP HG markedly suppressed joint inflammation and protect against bone destruction in arthritic rats.

This combination approach of PEITC and MTX (DD NP HG) synergistically improved anti-arthritic activity and reduced the adverse side effects *in vivo.*

## 1. Introduction

Rheumatoid arthritis (RA) is a progressive, chronic inflammatory condition of autoimmune nature characterized by synovitis, infiltration of inflammatory cells that cause cartilage degradation, bone erosion, and decalcification around the joints (Yin et al., 2020). With disease progression, joint deformities, and the related systemic symptoms lead to the loss of joint function and disability, reducing the life expectancy of RA patients (Ma et al., 2022). Although the pathophysiology of the disease is unknown, alleviating discomfort produced by joint swelling and preventing future cartilage loss has become an essential part of RA treatment (Yin et al., 2020). Currently, the available primary choices for the treatment of RA are opioids, cyclooxygenase-2 (COX-2) inhibitors, non-steroidal anti-inflammatory drugs (NSAIDs), disease-modifying anti-rheumatic drugs (DMARDs), and analgesics (Seo et al., 2022). However, these drugs are only marginally effective for short-term relief of symptomatic pain and inflammation. Furthermore, chronic use of DMARDs, NSAIDs, COX-2 inhibitors, corticosteroids, and opioids usually causes severe side effects in the gastrointestinal (GI) tract, liver, kidney, heart, and brain (Gerwin et al., 2006).

Natural isothiocyanates (ITCs) from vegetables have drawn a lot of research focus for the past two decades to treat and prevent cancer. PEITC is a member of the natural isothiocyanate family, a health-promoting molecule found in many cruciferous vegetables (Cang et al., 2014). One of the most studied natural ITCs, PEITC possesses significant antioxidant, anti-inflammatory, and anticancer properties (Hu et al., 2007; Shoaib et al., 2021). In addition to their many biological roles, PEITCs also play a crucial role in regulating osteoclastogenesis, a crucial process in conditions like osteoporosis and rheumatoid arthritis that affect the bones and joints (Murakami et al., 2007). The therapeutic application of PEITC is restricted by its poor aqueous solubility, instability, and low bioavailability, although cellular and animal studies significantly impact various disease conditions such as cancer and RA (Choudhary et al., 2020; Mohanty et al., 2020; Wang & Bao, 2021).

Methotrexate (MTX), the first-line therapy for RA, appears to have anti-inflammatory, anti-angiogenic, and analgesic properties (Wu et al., 2021). MTX, however, has poor aqueous solubility, poor pharmacokinetics, a narrow therapeutic time window, and nonspecific tissue distribution (Lyu et al., 2021), all of which lead to a significant risk of severe adverse effects, including bone marrow suppression, hepatotoxicity, nephrotoxicity, and severe neutropenia (Li et al., 2022). Direct intra-articular administration of MTX is also available to maximize the drug concentration at the target site and to limit systemic side effects. However, the fast clearance of MTX from the site renders it ineffective in reducing persistent inflammatory responses (Seo et al., 2022).

To address this limitation, it would be ideal for developing an injectable hydrogel depot formulation that can release and maintain the drug(s) concentrations for extended periods in articular joints. In RA research, thermosensitive injectable hydrogels have gained much attention among the hydrogel-based carrier systems since they have shown improved drug characteristics (solubility, stability, and drug bioavailability) and can be injected directly into inflamed joints (Yin et al., 2020). Thermosensitive hydrogels (TH) are smart drug delivery systems that remain in solution at room temperature and can transform into a gel at a higher temperature. Such TH are ideal as a depot drug delivery system as a transformation of the hydrogel from solution (at 25 °C) to gel (37 °C) ensures injectability, slow-release, and local delivery properties that can help obtain high local drug concentrations while lowering dose frequency and reducing systemic toxicity (Zhang et al., 2021).

Sodium alginate (SA) is a natural polysaccharide from a marine source widely employed in the food industry and, more recently, in biomedicine. SA and its hydrogels have been extensively explored in biomedical applications, either alone or in combination with other materials, primarily for drug delivery, tissue regeneration and wound healing, and three-dimensional (3D) printing. For tissue engineering, SA-based biomaterials have been extensively explored to treat soft and hard tissues, including skin, heart, bone, and cartilage (Neves et al., 2020). Pluronic F-127(PF-127, also known as poloxamer 407), is an amphiphilic triblock copolymer that exhibits reversible temperature-sensitive properties. Due to these unique properties, PF-127 is widely used as an injectable and can further facilitate a safe and sustained-release reservoir of a wide range of drugs, including hydrophilic and hydrophobic drugs *in situ* (Dumortier et al., 2006). The pluronic-based *in situ* injectable gelling systems have demonstrated significant promise as a drug delivery platform for numerous tissue engineering applications, including chondrogenesis and bone repair (Liu et al., 2020). However, due to the high porosity and limited mechanical strength, the pluronic hydrogel degrades quickly, making it unsuitable for prolonged drug administration. This drawback is significantly minimized by combining pluronic with additional biomacromolecules (chitosan, dextran, and sodium alginate) as composite hydrogels (Ur-Rehman et al., 2011).

Nanodelivery systems of therapeutic agents are an excellent approach for prolonged release/activity by increasing the residence time of the drug at the inflamed joints. Composite systems of various carriers, such as a nanocomposite hydrogel made of various nanoparticulate systems (i.e. micelles, nanoemulsion, and polymeric nanoparticles) and hydrogel, have the potential to expand therapeutic windows, boost bioavailability, and overcome the drawbacks of single drug delivery systems (Janakiraman et al., 2018).

The current study aims to develop a safe and biocompatible dual-drug loaded (MTX and PEITC) nanocomposite injectable hydrogel drug delivery system that, when administered IA, releases the drugs for a prolonged period alleviating the chronic inflammation and helps in the recovery of the stressful RA condition. To address the toxicity and poor solubility of both the drugs (MTX and PEITC), a nanocarrier (composed of nanoemulsion and nanoparticles) was utilized to encapsulate and deliver the drugs. PEITC occurs in oil form, which makes it an ideal choice for formulation as an oil-in-water (o/w) nanoemulsion, easing administration and mitigating downsides, including poor aqueous solubility, low stability, and limited bioavailability (Li et al., 2015). On the other hand, nanoencapsulation of MTX with poly (lactic-co-glycolic acid) (PLGA), the nanoparticles can target the inflamed joint by reducing the intrinsic toxicity of the drug, minimizing fast clearance, sustained drug release and improving drug retention (Liu et al., 2019). Dual-drug nanoparticles loaded hydrogel (DD NP HG) as a smart injectable depot was constructed by loading both the nanocarriers (MTX NP and PEITC NE). This combination in a hydrogel results in prolonged exposure of both drugs at the articular joints, that can facilitate its synergistic activity in promoting recovery from RA condition. The anti-arthritic efficacy of DD NP HG was examined using adjuvant-induced arthritis (AIA) in a rat model. Here, combining nanotechnology with materials science resulted in a sustained release of the nanomedicines resulting in the better therapeutic efficacy of drugs in the joints ensuring relief in the stressful RA condition.

**Scheme 1.**
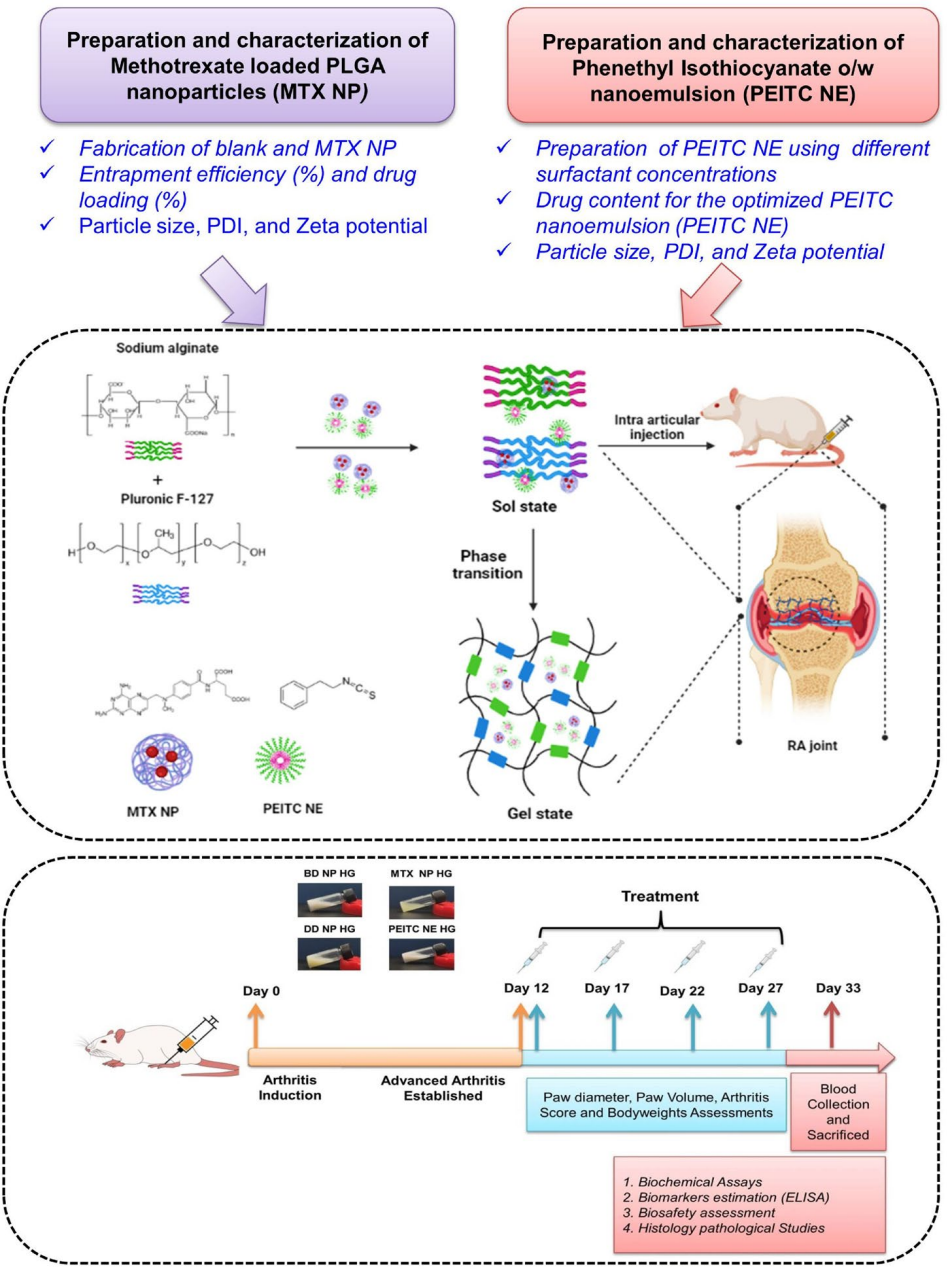
A schematic illustration of the preparation and a detailed combinatorial treatment schedule for treating RA using dual-drug nanoparticles loaded hydrogel (DD NP HG). Created with Biorender (biorender.com).

A schematic illustration of the fabrication of the smart hydrogel co-delivery system is presented in (Scheme 1). Here, the different steps involved in the preparation and characterization of nano-encapsulated drugs (MTX and PEITC) are shown with a detailed combinatorial treatment schedule for evaluating the therapeutic efficacy of the dual-drug nanoparticles loaded hydrogel (DD NP HG) as an injectable depot for treating RA.

## 2. Materials and methods

A detail of experimental procedures is provided in the supporting information file.

## 3. Results and discussion

### 3.1. Preparation of MTX-PLGA loaded nanoparticles (MTX NP)

Despite exhibiting good anti-arthritic activity, the clinical usage of Methotrexate (MTX) is limited due to its undesirable properties (poor solubility, low bioavailability, and several side effects). In this study, a nanoprecipitation method (using solvent evaporation) was employed to overcome a few physicochemical limitations of MTX by loading into a biodegradable PLGA polymer matrix. For optimization purposes, three different batches of MTX NPs were prepared using 0.1% (w/v) PF-127 (as surfactant) with varying concentrations of PLGA (80, 120, 150 mg), yielding an entrapment efficiency of **53.73 ± 4.83%** (2.79 ± 0.25 mg), **64.60 ± 2.34%** (3.36 ± 0.12 mg) and **78.45 ± 3.10%** (4.08 ± 0.16 mg) respectively (Table 1).

**Table 1.** Different polymer compositions with different % EE and % DL.

Batch no	PLGA polymer composition (mg)	Amount of drug (mg)	Solvent mixture (Acetone: DMSO)	PF-127 % (w/v)	% EE	% DL
**I**	80	5.2	9:1	0.1	53.73 ± 4.83	2.66 ± 0.24
**II**	120	5.2	9:1	0.1	64.60 ± 2.34	2.31 ± 0.08
**III**	150	5.2	9:1	0.1	78.45 ± 3.08	2.33 ± 0.09

It was observed that the increase in PLGA concentration increased the drug’s %EE (% encapsulation efficiency) as the increase in viscosity of the organic phase hampered the diffusion of the drug from the organic phase to the aqueous phase. Here, the undiffused lipophilic drug might be entrapped in the matrix of alkyl chains of PLGA with weak interactions and released slowly from the polymeric matrix (Ray et al., 2015). Among the prepared formulations, **batch no-III** (PLGA- 150 mg) with a greater % EE of the drug was further utilized to fabricate DD NP HG.

### 3.2. Preparation and optimization of PEITC o/w nanoemulsion (PEITC NE)

An oil-in-water nanoemulsion of PEITC was prepared by dissolving it in olive oil to form the oil phase. Olive oil, a model oil with mild anti-inflammatory activity, may also aid in the potentiation of treatment efficacy. In the current preparation, the aqueous phase (72%) and oil phase (18%) were kept constant, varying only the surfactant ratios. To obtain a stable emulsion, two non-ionic surfactants, span-80 and tween-80, were used as surfactant mixtures (Li et al., 2015). Both stirring and probe sonication were employed to reduce the droplet size to an optimum range. A total of eight different batches were prepared by altering the surfactant concentrations based on the HLB value. The HLB value of the surfactant mixtures between 4 and 5 exhibited higher stability than other batches (**batch-7** HLB of 4.62 and **batch-8** HLB of 4.3) where no creaming or phase separation was observed, as shown in Supporting Figure S1. Therefore, these two batches (**7 & 8**) were selected and, with further optimization, yielded an improved (lower) particle size of 240.67 ± 4.75 nm (**batch-8**) and 303.5 ± 5.12 nm (**batch-7**), as shown in Supporting Table S1. This may be due to increased lipophilic surfactant (Span-80) that might help in the uniform dispersion of highly lipophilic PEITC into the aqueous phase. The drug content in the finally optimized formulation of PEITC NE (**batch-8)** was found to be **83.16 ± 10.87%** (33.4 ± 4.35 mg) (Supporting Table S1).

**Figure 1.**
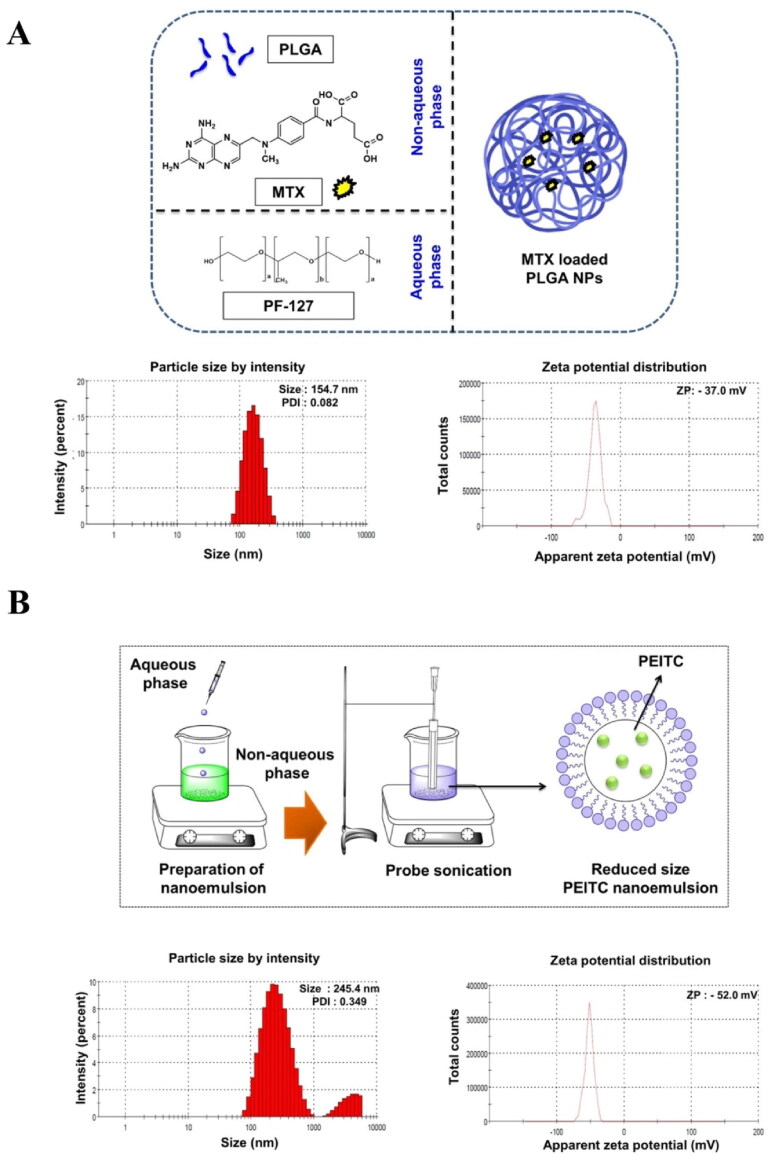
Characterization of nanoparticle (NP) and nanoemulsion (NE). The particle size (PS), polydispersity index (PDI), and zeta potential (ZP) of (A). MTX NP and (B). PEITC NE were determined using dynamic light scattering (DLS). The values are represented as mean ± SD of triplicate measurements.

### 3.3. Particle size characterization of drug-loaded nanoparticle formulation

Particle size characterization of both the formulations (MTX NP and PEITC NE) by DLS confirmed a monodisperse, nanometer-sized preparation with good zeta potential (Table 2 and Figure 1A and B). The optimized MTX NP had a negative surface charge (ave ZP of −34.50 mV), narrow size distribution with a mean diameter of **151.8 ± 2.65 nm**. On the other hand, PEITC NE had a negatively charged surface (Z = -50.73 mV) with an average size of **240.67 ± 4.75 nm.** Finally, the optimized uniformly dispersed drug nanoparticles (MTX NP and PEITC NE) were suitably loaded into the alginate-pluronic hydrogel system to prepare as an injectable for intra-articular administration (Danaei et al., 2018; Trujillo-Nolasco et al., 2019).

**Table 2.** Particle size characterization of PEITC NE and MTX NP formulations. Particle size (PS), polydispersity index (PDI), and zeta potential (ZP).

Sl.no.	Name of the formulation	PS (nm)	PDI	ZP (-mV)
**1**	**MTX NP**	151.8 ± 2.65	0.099 ± 0.015	−34.5 ± 2.29
**2**	**PEITC NE**	240.67 ± 4.75	0.351 ± 0.003	−50.73 ± 2.02

### 3.4. Fabrication and validation of dual-drug nanoparticles loaded hydrogel

Following the optimization studies, two SA-hydrogels single NP-loaded with MTX NP (MTX NP HG) or PEITC NE (PEITC NE HG) and a dual-drug loaded hydrogel loaded with MTX NP and PEITC NE were prepared with the composition as given in the Table 3. The three individual HG formulations were utilized for further studies, as described below.

**Table 3.** Different compositions of SA and PF-127 for the preparation of *in-situ* hydrogels.

Batch no.	Name of the hydrogel	SA composition (%) (w/v)	PF-127 composition (%) (w/v)
**I**	MTX NP HG	1	19
**II**	PEITC NE HG	1	12
**III**	DD NP HG	1	12

### 3.5. Characterization of dual-drug nanoparticles loaded hydrogel

#### 3.5.1. FTIR and FESEM studies

FTIR studies were performed to observe any peak shifts due to chemical alterations in the drugs (MTX and PEITC) and the polymers/excipients (olive oil, span-80, tween-80, PLGA, SA, and PF-127) utilized in the formulation of nanoparticular hydrogels.

**Figure 2.**
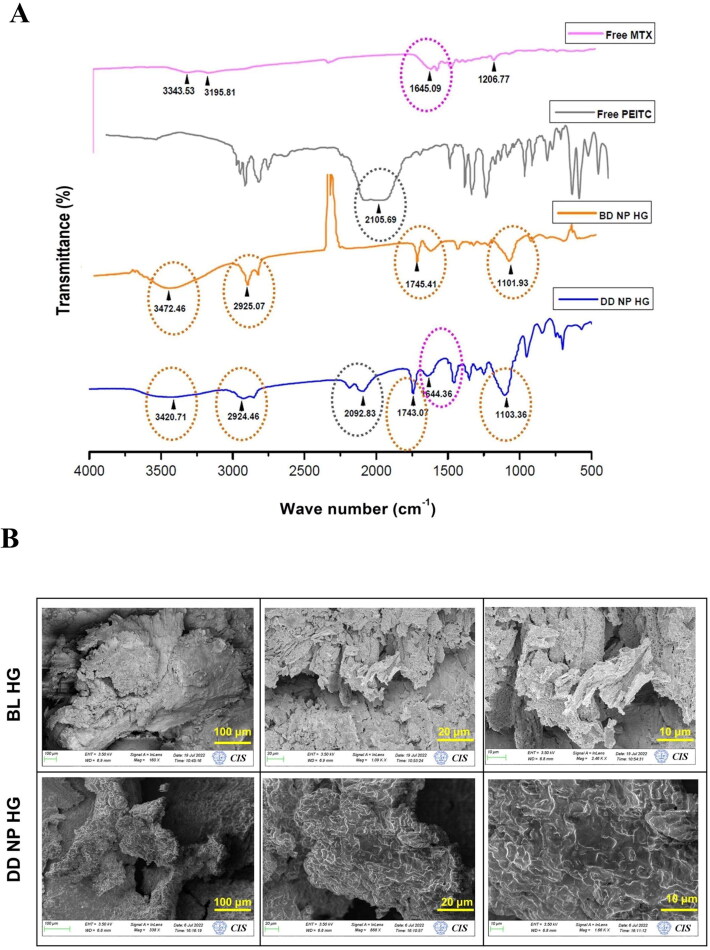
Characterization of dual-drug nanoparticles loaded hydrogel. (A). FTIR spectra of Free MTX, Free PEITC, BD NP HG, and DD NP HG. (B). FESEM images represent the morphological structure of BL HG and DD NP HG. Scale bars represent 100, 20, and 10 μm. FTIR: Fourier-Transform infrared spectroscopy, FESEM- Field Emission Scanning Electron Microscopy.

The FTIR spectra (Figure 2A) of different drug-loaded hydrogels are compared with the peaks of BD NP HG and their respective free drugs to ensure drug incorporation and verify covalent modifications if any. The following are the similar functional group characteristic peaks observed between BD NP HG and DD NP HG gel, respectively: i) A hydroxyl (–OH) group stretching vibration was observed at 3472.46 cm^−1^ (BD NP HG) and 3420.71 cm^−1^ (DD NP HG) that might be due to PLGA polymer (in MTX NP) or from alginate (Dalal et al., 2021). ii) The –CH group stretching vibration at 2925.07 cm^−1^ (BD NP HG) and 2924.46 cm^−1^ was assigned to the alkyl chains of alginate/pluronic (Karolewicz et al., 2017; Dalal et al., 2021). iii) The characteristic –C = O (COOH) peak at 1745.41 cm^−1^ (BD NP HG) and 1743.07 cm^−1^ (DD NP HG) was assigned to the acidic group in PLGA (Gao et al., 2017) or olive oil containing several acids (Wang et al., 2020). iv) The –C-O stretching vibration at 1101.93 cm^−1^ (BD NP HG) and 1103.36 cm^−1^ (DD NP HG) relates to the ether chains of the PF-127 used in the hydrogel formulation (Karolewicz et al., 2017).

The principal absorption peaks related to drugs (PEITC and MTX) showed an isothiocyanate (-N=C=S) peak of PEITC at 2105.69 cm^−1^ (Coscueta et al., 2021) and MTX at 3343.53 cm^−1^ confirming the presence of the -OH group, and 1645.09 cm^−1^ corresponding to the –C=C group (Jang et al., 2019). A comparison of the FTIR spectra of Free PEITC with DD NP HG FTIR showed a distinctive peak at 2092.83 cm^−1^, corresponding to the –N=C=S group, and a peak at 1644.36 cm^−1^ for the –C=C group. This data confirmed that PEITC NE and MTX NP are successfully incorporated with weak interactions into the hydrogel matrix.

FESEM studies were performed on the freeze-dried and gold sputter-coated samples of BL HG and DD NP HG hydrogels to investigate the cross-linked structure of the hydrogels. The images for both the hydrogel samples showed a macroporous structure with no apparent differences; however, a more complex 3D microstructure was observed in DD NP HG than in the BL HG (Figure 2B). The representative FESEM images of DD NP HG shown in Figure 2B displays a better-interconnected network with more distinctive pores than that of BL HG. This result shows that the fabricated dual-drug loaded nanoparticles inside the hydrogel will be relatively more favorably distributed within the DD NP HG hydrogel structure and could retain a sustained and controlled drug release due to the more interconnected porous structure.

#### 3.5.2. Drug content

Following the qualitative validation of the incorporation of both drugs (MTX and PEITC) into the hydrogel, a centrifugation method was performed to assess the individual drug content (%) in the different hydrogel formulations. As shown in Table 4, the %drug content for different hydrogel formulations ranged between **81%** and **90%.**

**Table 4.** Drug content (%) of different hydrogel formulations.

Batch no	Name of the hydrogel	Drug content (%)	Amount of drug loaded into the hydrogel (mg/mL)
**I**	MTX NP HG	90.87 ± 1.90	3.63 ± 0.076
**II**	PEITC NE HG	85.63 ± 3.36	34.25 ± 1.35
**III**	DD NP HG (MTX)	88.24 ± 9.27	3.53 ± 0.37
**IV**	DD NP HG (PEITC)	81.75 ± 3.50	32.7 ± 1.4

#### 3.5.3. Solubility studies

Solubility studies were performed to determine the efficiency of nanoparticles or emulsions loaded into the hydrogel to solubilize the poorly soluble drugs. In the current study, both the drugs MTX and PEITC are hydrophobic, which is a major drawback for their clinical utilization. As shown in Supporting Figure S2, % aqueous solubility of Free MTX and MTX in DD NP HG was found to be **0.36 ± 0.29%** (1.46 ± 1.17 µg/mL) and **72.96 ± 4.88%** (29.18 ± 1.95 µg/mL) respectively. On the other hand, PEITC, which is immiscible in water, showed a considerable increase in its aqueous solubility (%) up to **73.13 ± 8.86%** (29.25 ± 3.55 µg/mL) in the DD NP HG (PEITC) hydrogel formulation.

The solubility studies showed that the nanocomposite hydrogel system considerably improved the solubility of both loaded drugs (MTX and PEITC). Compared to free form, this higher solubility may increase the effectiveness in treating arthritic conditions by potentiating the release of both drugs in their nanoparticulate forms.

#### 3.5.4. Sol-gel phase transition studies

Typically, it is anticipated that the thermosensitive hydrogels will continue to exist in the sol state at a temperature between 4 °C to 25 °C before gradually transitioning to a gel state at physiological body temperature (37 °C). To properly construct a depot injectable, it is imperative to consider the sol-gel transition period, mainly dependent on the temperature, polymer concentration (PF-127), and oil content. In the PF-127 structure (PEO-PPO-PEO), PPO chain forms are responsible for forming temperature-dependent packed micellar microstructure. At optimum concentrations and temperatures, PEO chains often overlap adjacently with the packed micellar structures, giving rise to a thermoreversible gelation property.

**Figure 3.**
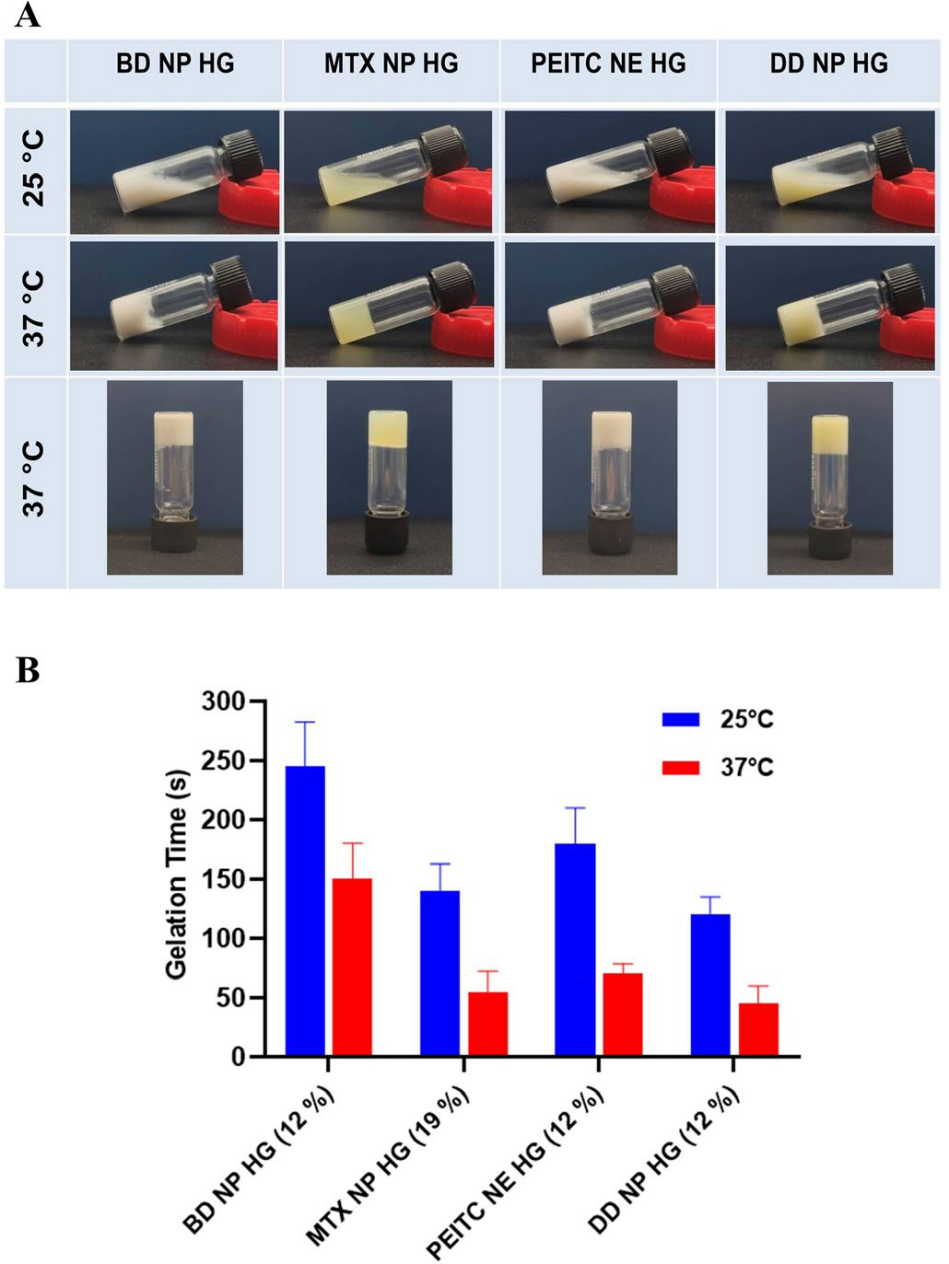
Sol-gel phase transition characteristics and determination of gelation time (A). Representative image of the sol-gel phase transition characteristics of different hydrogel formulations (BD NP HG, MTX NP HG, PEITC NE HG, and DD NP HG ). Phase transition with an increased temperature. (B). The gelation time of the different hydrogel formulations (BD NP HG, MTX NP HG, PEITC NE HG, and DD NP HG) was measured at 25 °C and 37 °C. Data shown in mean ± SD (*n* = 3).

In the current study, sol-gel transition studies were carried out at two different temperatures, i.e. 25 °C and 37 °C, respectively, to verify the thermosensitivity of the *in situ* hydrogels. All prepared hydrogels underwent a sol-to-gel transition from 25 °C to 37 °C (Figure 3A). The drug-incorporated nanoparticle considerably affected the gelation time at 25 °C and 37 °C. As observed in Figure 3B, the time for gelation for BD NP HG was relatively higher **(245 ± 37.7 and 150 ± 30 s)** in comparison to the other hydrogels; MTX NP HG **(140 ± 22.9 s and 55 ± 17.3 s),** PEITC NE HG **(180 ± 30 s and 70 ± 8.7 s) and** DD NP HG **(120 ± 15 s and 45 ± 15 s)** at 25 °C and 37 °C respectively. The time difference in the gelation at 25 °C and 37 °C was found suitable, giving ample time for administration of the formulation into the site and its gelation (Figure 3B).

The adsorption of PPO groups of PF-127 at the interface of the oil droplet makes it more hydrophobic, and these highly hydrophobic groups further form mixed micelles responsible for its thermosensitive behavior. Hence, the nanoemulsion-loaded hydrogels exhibit thermoreversible characteristics even at lower concentrations of PF-127 (Hashemnejad et al., 2019). It was observed that with an increase in the PF-127 concentration (> 12% w/v), a hard gel was formed that prevented the gel from passing through the syringe even at a lower temperature (< 10 °C), resulting in poor injectability. Hydrogel-containing nanoemulsion constructed with 12% (w/v) of PF-127 has a considerable gelation time difference between 25 °C and 37 °C. Hence 12% (w/v) PF-127 concentration is preferred in the preparation of the hydrogel-containing nanoemulsion (PEITC NE HG and DD NP HG) to ensure good injectability and thermosensitive properties.

#### 3.5.5. *In vitro* degradation studies

**Figure 4.**
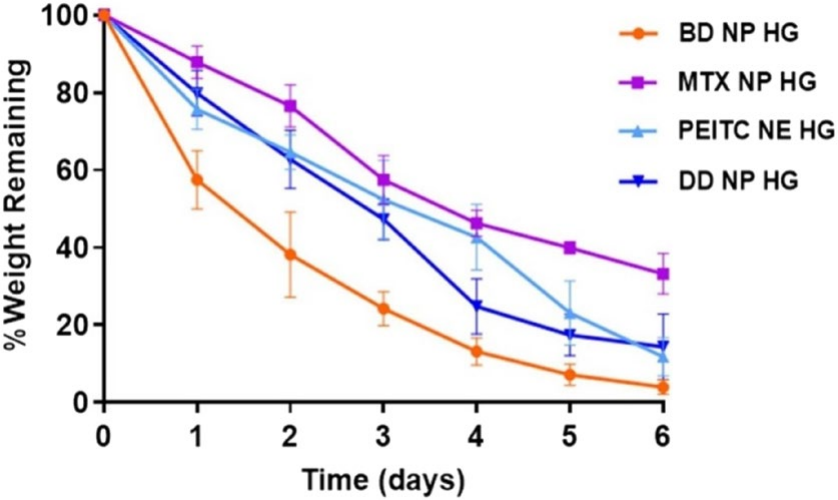
*In vitro* degradation properties of dual-drug nanoparticles loaded hydrogel. The rate of degradation (%) of the hydrogels at 37 °C was investigated by estimating the % amount of residual hydrogels at various time intervals up to 6 days. Data expressed as mean ± SD (*n* = 3).

An *in vitro* degradation study of all the hydrogels was carried out *in situ* as a function of time in PBS (pH 7.4) at 37 °C for 6 days to predict its *in vivo* biodegradability or stability (Pankongadisak & Suwantong, 2019). The degradation kinetics of the different hydrogels (MTX NP HG, PEITC NE HG, and DD NP HG) and the % remaining weight showed more than 67% degradation for up to 6 days, indicating sustained bio-degradation (Figure 4). The final % weight of remaining hydrogel MTX NP HG, PEITC NE HG, and DD NP HG was determined to be **33.18%, 11.78%,** and **14.32%**, respectively, while the BD NP HG showed higher degradation (remaining of **3.86%**).

#### 5.6. Injectability studies

The *in vivo* performance of the hydrogel as an injectable can be studied *in vitro* using a physiologically simulated condition (37 °C). The different hydrogel formulations moved freely into the syringe. Upon injection into PBS (at 37 °C), the contents moved smoothly out of the needle forming an uninterrupted thread-like soft gel settling at the bottom of the vial without any phase separation or clumps, indicating a homogenous and stable preparation suitable as a thermoresponsive injectable. (Supporting Figure S3).

#### 3.5.7. Rheological characteristics

Determining the rheological characteristics of injectable hydrogels is essential for understanding the behavior of a hydrogel following injection and also for understanding the structural behavior of the formulation by measuring the viscoelastic property as a function of frequency, temperature, and stress/strain. The most significant visco-elastic rheological parameters considered for its application in the biomedical area are complex viscosity (η*), storage modulus (G’), and loss modulus (G"). Complex viscosity measures the ability of the gel to resist shearing forces within an injection site during injection. In contrast, storage modulus (G’) and loss modulus (G") differences measure the sol-gel conversion of the injectable hydrogel (Alonso et al., 2021).

**Figure 5.**
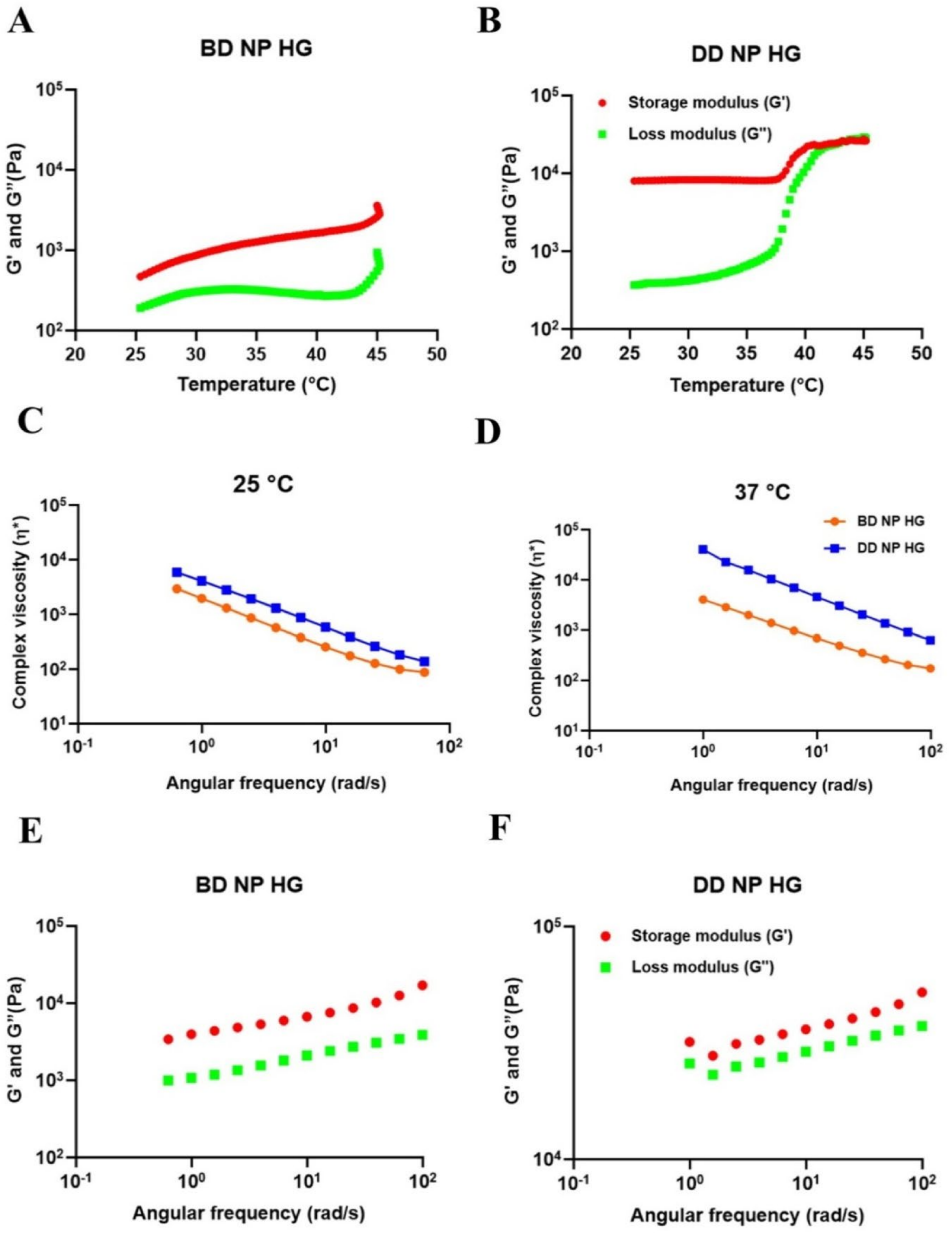
The rheological properties of (A). BD NP HG and (B). DD NP HG. Complex viscosity versus angular frequency for BD NP HG and DD NP HG at (C). 25 °C and (D). 37 °C. Storage modulus (G’) and loss modulus (G’’) of the (E). BD NP HG and (F). DD NP HG hydrogels on the angular frequency at 37 °C.

In order to measure the temperature-dependent viscoelastic property of the hydrogel, the temperature sweep test was performed at the constant frequency and strain rate. The rheological parameters of the BD NP HG and DD NP HG, namely G’ (storage) and G” (loss) modulus, have been illustrated as the function of temperature. As shown in Figure 5A and B, all the rheological parameters depend highly on the temperature. From the heating cycle, 25 °C was determined to be the sol-gel transition temperature for both hydrogel formulations. The BD NP HG and DD NP HG show a gel-like behavior at the beginning of the heating cycle (after 25 °C) as the storage modulus is higher than the loss modulus. In the gel state, the storage modulus and loss modulus values of BD NP HG and DD NP HG indicate that both are mechanically stable. The sample DD NP HG shows a sudden increase in the storage modulus value at 37 °C during the heating cycle, whereas the sample BD NP HG shows a gradual increase in the modulus during the heating cycle. Thus, the sample DD NP HG has a higher level of gel structure above 37 °C.

The frequency dependence (and equivalently, the time dependence) of a material’s viscoelastic characteristics is commonly measured using frequency sweeps. High and low frequencies, respectively, generally represent short-time and long-time scales. The sinusoidal stress response is used to calculate the dynamic moduli G" (loss) and G’ (storage). The complex viscosity, frequently used in place of the complex modulus, is calculated as the complex modulus divided by the applied frequency. Frequency tests are typically conducted in the linear range (small strain); in this case, the results are independent of the applied strain. Figure 5C and D shows the complex viscosity of the gel measured as a function of frequency at 25 °C and 37 °C. The hydrogel shows the shear-thinning behavior as the gel is subject to a higher rate of deformation - which indicates that the complex viscosity (η*) is decreasing with the increasing rate of deformation (i.e. angular frequency). This suggests that the hydrogels were highly shear thinning and are suitable as an injectable for animal *in vivo* studies. The hydrogel DD NP HG shows a higher complex viscosity than the hydrogel sample BD NP HG at both temperatures.

The storage modulus (G’) and loss modulus (G") of the hydrogel changed with angular frequency. From the results in Figure 5E and F, G′, and G" increased obviously at 37 °C, with the angular frequency increasing. By observing data, DD NP HG showed increased levels of both G′ and G" compared to BD NP HG. This indicates that the incorporation of drugs in the hydrogel leads to the strength of hydrogel increased obviously and enhanced mechanical stability. Hence, G’ was always greater than G", which is a typical gel-like flow behavior when it is subjected to a higher frequency (i.e. rate of deformation).

The improved rheological parameters of DD NP HG might occur due to strong inter-micellar interactions between the drug-loaded nanoparticulate system inside hydrogels with SA and PF-127 in the hydrogel.

### 3.6. *In vitro* drug release studies

*In vitro* dissolution studies were conducted to evaluate the rate and quantity of drug release, which may have a more substantial impact on treating disease, prior to the *in vivo* assessment studies for a particular drug formulation (dose frequency and assessment). In most of the studies, it is believed that improved solubility and dissolution with sustained drug release behavior may enhance the treatment efficiency. An additional advantage of the *in vitro* release study is that it helps to decide the frequency of dosing in the *in vivo* treatment studies.

**Figure 6.**
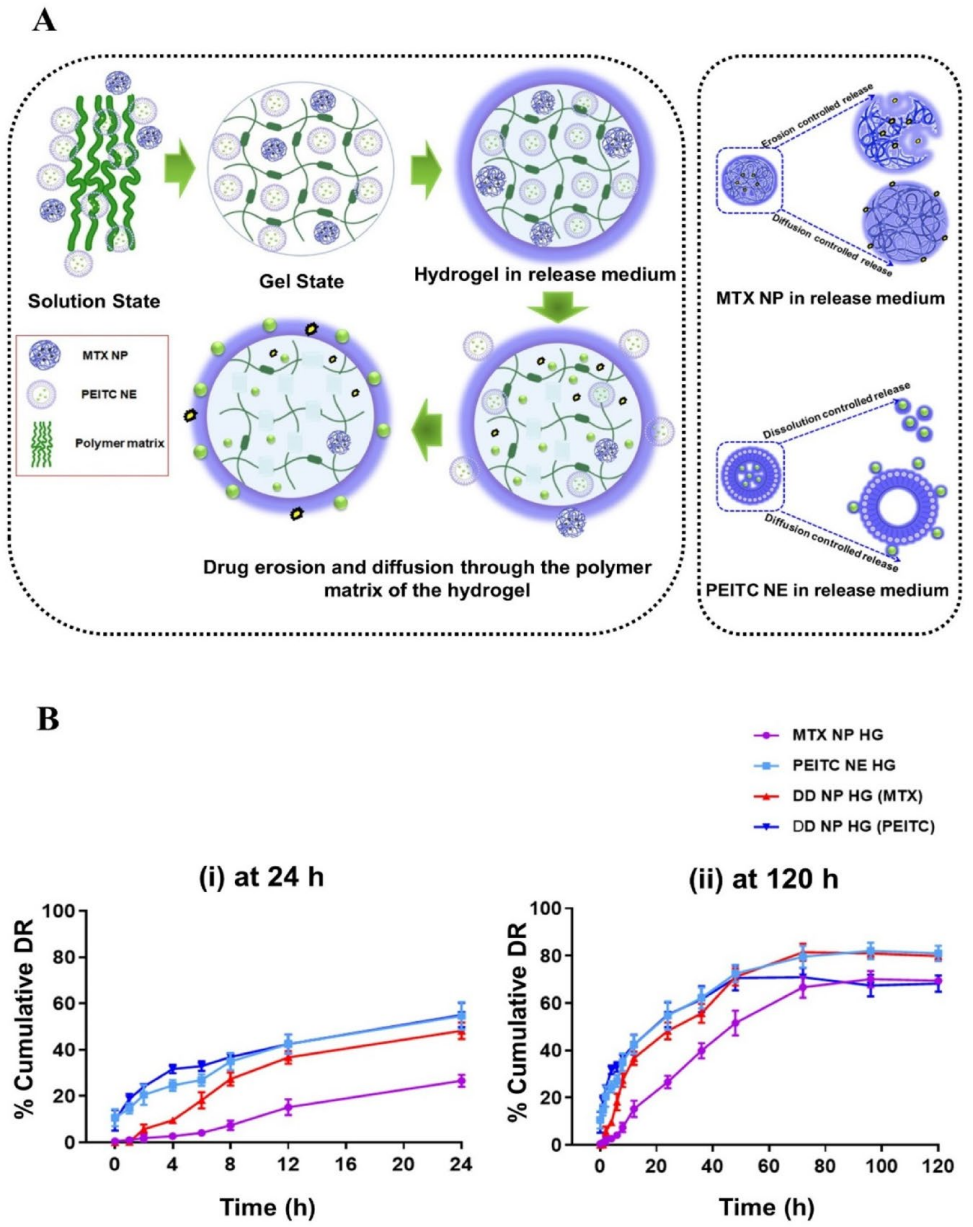
The drug release properties of dual-drug nanoparticles loaded hydrogel in vitro. (A). Schematic representation of drug release mechanism of DD NP HG. (B). % MTX and PEITC release from the hydrogel vs. time (h) at pH 7.4 at 37 °C (i). 24 h and (ii). 120 h was measured. *purple* color: MTX release rate from MTX NP HG, *light blue* color: PEITC release rate from PEITC NE HG, *red* color: MTX release rate from DD NP HG, and *navy blue* color: PEITC release rate from DD NP HG. Results are presented as mean ± SD (n = 3). Created with Biorender (biorender.com)

In the current study, all the hydrogels showed > 68% drug release sustaining up to 120 h, shown in Figure 6B (ii). Initially, the amount of PEITC release from both the hydrogels was 55% (DD NP HG) and 54% (PEITC NE HG) in 24 h, and a sustained release behavior was observed upto 120 h (PEITC NE HG −81% & DD NP HG − 68%) . In contrast, MTX released from the hydrogel was 48% in DD NP HG and 26% in MTX NP HG, respectively, in 24 h; over time, an extended-release was studied upto 120 h in both the hydrogels (MTX NP HG − 69% & DD NP HG- 79%). The variable release rate of MTX may be due to the slow erosion or degradation of the rigid hydrogel network surrounding the MTX NP, which is observed in the *in vitro* degradation studies, or another reason may be the nanoemulsion potentiates the solubility of MTX in the combinational nanogel and further aids in the increased dissolution of the MTX (Van Hemelryck et al., 2019). Owing the saturation of lipophilic drug in the buffer solution or adsorption of the drug to the dialysis membrane may be an obstacle to preventing 100% of the drug release. This may be overcome by increasing the sink condition (Ahsan et al., 2020).

In summary, hydrogels not only increased the solubility and dissolution but also sustained the release of both drugs for 120 h, which is ideal for the delivery of drugs continuously in the inflamed joints for a prolonged period.

### 3.7. In vitro drug release kinetics

The *in vitro* drug release kinetics results suggested that MTX from DD NP HG followed the Korsmeyer-Peppas model for 24 h and later by the Higuchi model. In contrast, MTX NP HG followed zero order initially (for 24 h) and followed by the Korsmeyer-Peppas model. In the case of PEITC, it followed a similar mechanism pattern from both the hydrogels (DD NP HG and PEITC NE HG), initially released by the Korsmeyer-Peppas model for 24 h and then continued with the Higuchi model (Supporting Figure S4, Table S2). The Higuchi model represents that the drug is embedded inside the hydrogel matrix and slowly diffuses out by both the diffusion and erosion mechanism. Solvent or dissolution media enters the matrix, then dissolves and transfers the drug from inside to outside the membrane by erosion and diffusion-controlled mechanisms.

The Korsmeyer model depicts anomalous drug diffusion from the hydrogel network. The zero-order model represents the controlled release of the drug diffusion from the hydrogel network.

### 3.8. Stability studies for different hydrogel formulations

The hydrogels exhibited good stability at 4 °C with negligible differences in % drug content (% DC) shown in Supporting Table S3.

### 3.9. Anti-arthritic effects of the dual-drug nanoparticles loaded hydrogel

#### 3.9.1. Effects of dual-drug nanoparticles loaded hydrogel on arthritis parameters in FCA-induced arthritic rats

**Figure 7.**
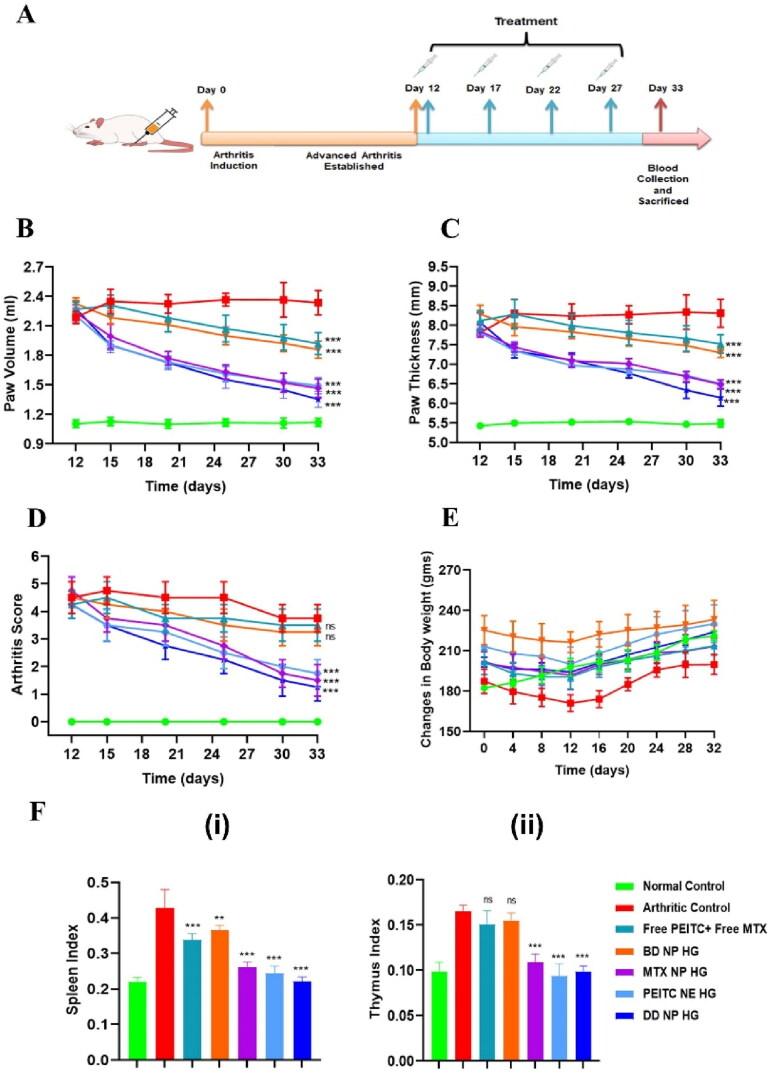
*In vivo* anti-arthritic effect of the dual-drug nanoparticles loaded hydrogel in FCA-induced RA rats. (A). Timeline for the dual-drug nanoparticles loaded hydrogel treatment in RA model rat (FCA-induced) with different treatment groups. (B). Changes in paw volume over time in the different treatment groups. (C). Changes in paw thickness over time in the different treatment groups (D). Arthritis scores for RA in rats on following treatment with different formulations. According to the standard scoring method, the average arthritis score was assessed by blind testing (scale of 0–4). (E). Changes in body weight in the FCA-immunized rats were measured from day 0-32  following treatment. (F). The index of (i) spleen and (ii) thymus on day 33 in different treatment groups of FCA-immunized rats. The index of the thymus and spleen were expressed as the ratio (mg/g) of thymus and spleen wet weight versus body weight, respectively. The data are represented as mean ± SD (*n* = 4). The values were statistically examined using one-way ANOVA test. Statistical significance: *p < 0.05, **p < 0.01, ***p < 0.001, and ns- non-significant. FCA: Freund’s adjuvant, Complete.

The therapeutic efficacy of combinational nanocomposite hydrogel was investigated in a chronic rat model of RA immunized by Freund’s Complete Adjuvant (FCA). After FCA immunization, the rats were randomly assigned into various experimental groups on day 12 and continued till day 33 as the end study point (see Figure 7A).

The changes in paw volume and thickness were the initial parameters observed to measure inflammation and swelling in adjuvant-induced arthritic (AIA) rats and the treated groups. Following FCA immunization, on day 12, the different AIA groups of rats received an IA injection of either PBS, Free PEITC + Free MTX, PEITC NE HG, MTX NP HG, BD NP HG, and DD NP HG at an interval of every four days for up to 27 days where changes in the arthritis score and swelling of the entire paw were observed and recorded (Figure 7B and C). Paw volume and paw thickness in the inflamed paws gradually increased in the PBS-treated rat as the disease progressed, while this progression was slower in the animals treated with the PEITC NE HG, MTX NP HG, and DD NP HG. Compared to the PBS-treated group, the PEITC NE HG and the MTX NP HG alleviated the symptoms of arthritis in rats. However, Free PEITC + Free MTX and blank HG loaded with blank NP failed to inhibit the disease progression, as there was no effect on the inflamed paw volume and thickness of the paws in these groups. The treatment with DD NP HG improved the arthritis symptoms (i.e. paw volume and paw thickness) to a greater extent among the groups (Figure 7B and C).

Hind paws of RA rats were scored for signs of arthritis (i.e. arthritis score) to monitor the disease severity and assess the prepared formulation’s effectiveness in treating RA. A modest amount of erythema and swelling was evident in the current AIA rat model on the 5^th^ day following FCA injection. On the 12^th-^day post-FCA injection, considerable swelling and erythema appeared in the injected hind paws indicating the full onset of RA. The AIA-rats were then administered with different formulations every four days, initiated on day 12 and ending on day 27. As depicted in Figure 7D, after treatment, groups treated with PEITC NE HG, MTX NP HG, or DD NP HG exhibited a reduction in the arthritis scores.

At the endpoint, i.e. the 33^rd^ day, the Free PEITC + Free MTX and BD NP HG treated groups showed a cumulative arthritis score of **3.50 ± 0.58** and **3.25 ± 0.50** and the arthritic control group with an arthritis score of **3.75 ± 0.50.** The rat treated with DD NP HG showed a marked reduction in arthritis score **(1.25 ± 0.50)** than the Free PEITC + Free MTX and BD NP HG treated group and the arthritic control group. PEITC NE HG and MTX NP HG treated animals had arthritis scores of **1.75 ± 0.50** and **1.50 ± 0.58,** respectively.

In arthritic rat models, reduction in body weight is an essential parameter to measure inflammation. Changes in the body weights were monitored for up to 32 days to measure FCA-induced inflammation and the effectiveness of treatment groups, as presented in Figure 7E. Here, the normal healthy control group showed an increased body weight than the arthritic control group. The body weight loss in FCA-induced rats might be attributed to restricted mobility, decreased intestinal absorption, and inflammatory response. After 32 days, compared to the arthritic control group, DD NP HG treated group showed improvement and significantly gained body weight. These findings imply that DD NP HG has effectively decreased arthritic-induced inflammation.

Assessing the ratio of primary immune organs (such as the spleen and thymus) is a vital indicator in relating an autoimmune condition (like RA) with its disease progression (Wang et al., 2019). As a result, alterations in the spleen and thymus indexes were assessed across all study groups. Figure 7F (i-ii) shows that the arthritic control group had a higher spleen and thymus index than the normal healthy control group. The spleen and thymus index in the treatment groups, PEITC NE HG, MTX NP HG, or DD NP HG, decreased significantly compared to the arthritic control group. Comparing the animals treated with Free PEITC + Free MTX and BD NP HG to the arthritic control group, no discernible differences were seen in the spleen and thymus indexes. Compared to other treatment groups, the group receiving DD NP HG treatment showed a remarkable improvement in the spleen index.

The trend observed comparing the increased paw volume and cumulative arthritis scores in arthritic rats demonstrate that the DD NP HG group helps to improve arthritis disease conditions more effectively than the PEITC NE HG and MTX NP HG groups, suggesting that the combined therapeutic effect of the two drugs in their nanoparticulate form was more effective than the therapeutic effect of the individual nano form of the drug itself.

#### 3.9.2. Effect of dual-drug nanoparticles loaded hydrogel on oxidative stress biomarkers in FCA-induced arthritic rats

The pathogenesis of RA is greatly influenced by oxidative stress. Reactive oxygen species (ROS), such as NO, H_2_O_2_, O_2_, and HOCl, are produced from various infiltrating activated immune cells. Different detoxifying enzymes, such as GSH, naturally shield the tissues from harmful ROS (Wruck et al., 2011). However, as RA progresses, these protective mechanisms gradually become reactive due to increased ROS production, which results in the peroxidation of lipids (formation of MDA), which worsens the inflamed joint tissues and exacerbates the disease situation (Zimmerman et al., 2017). Simultaneously neutrophil granule proteins, such as myeloperoxidase (MPO), are also detected in high amounts in the synovial fluid of the RA, which are responsible for joint destruction (Li et al., 2019).

**Figure 8.**
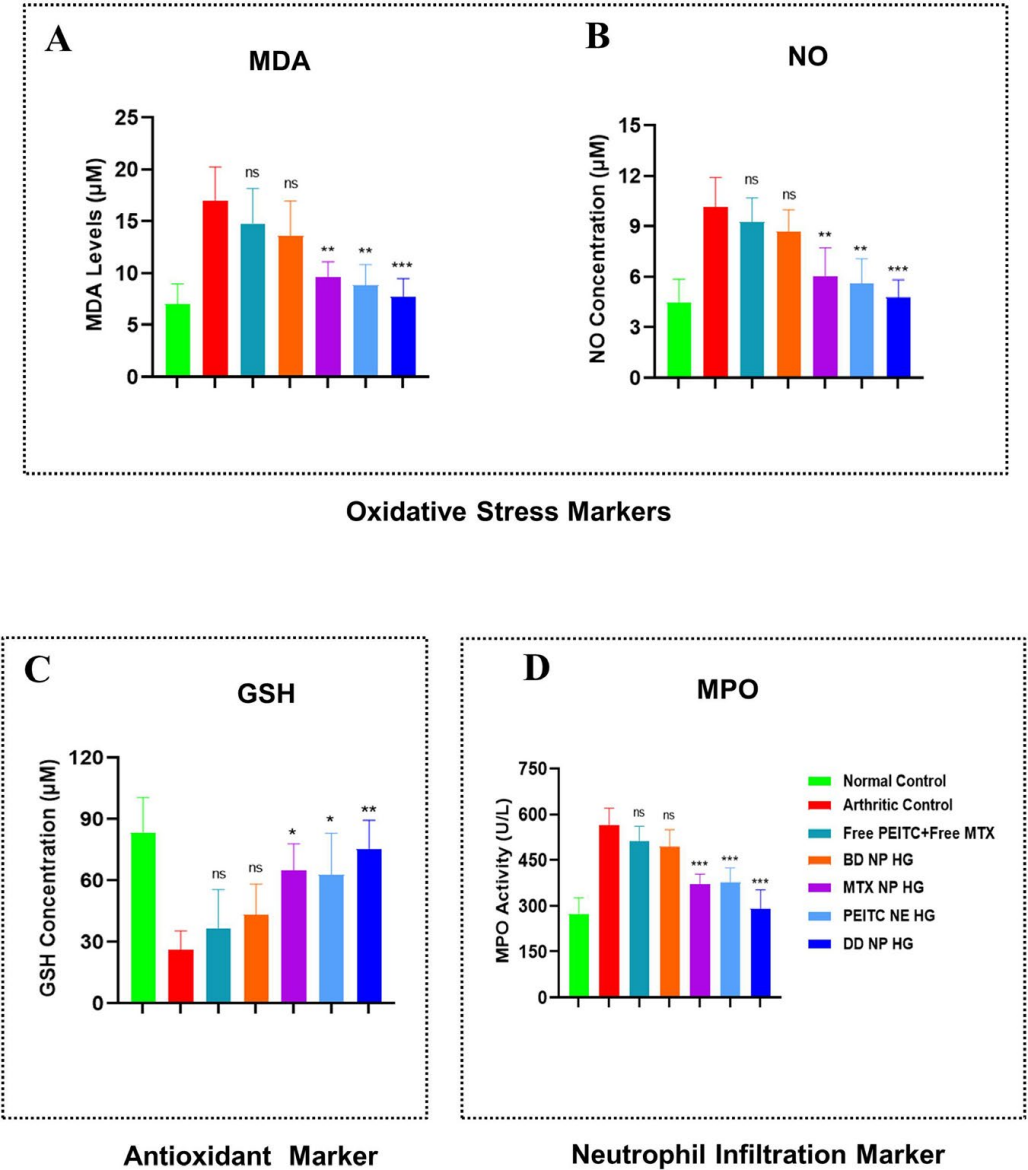
Evaluation of oxidative stress, antioxidant, and neutrophil infiltration markers in FCA-induced RA rats. Effect of dual-drug nanoparticles loaded hydrogel on serum concentration of (A). MDA (B). NO (C). GSH and (D). MPO. On day 33, the serum of the different treatment groups was collected and quantified. The data are represented as mean ± SD (*n* = 4). The values were statistically examined using a one-way ANOVA test. Statistical significance: *p < 0.05, **p < 0.01, ***p < 0.001 and ns-non significant. MDA: Malondialdehyde, NO: Nitric oxide, GSH: Glutathione, and MPO: Myeloperoxidase.

MDA levels were also detected in the serum as an indicator of lipid peroxidation following 33 days of FCA induction (Qindeel et al., 2020). A noticeable increment in the serum MDA levels was observed in rats subjected to AIA compared to normal healthy control groups. Figure 8A demonstrates that the arthritic control group displayed a **2.42**-fold increased MDA level than the normal healthy control group. Following treatment with PEITC NE HG, MTX NP HG, and DD NP HG, a substantial decrease in MDA level was noted. However, the levels of MDA formation were decreased by **1.93** and **1.77**-fold, respectively, by PEITC NE HG and MTX NP HG, and 2.20-fold further reduced this formation in the group treated with DD NP HG. Compared to the arthritic control group, the levels of MDA in the Free PEITC + Free MTX and BD NP HG groups decreased by **1.15**-fold and **1.25-**fold, respectively (Figure 8A).

The number of total nitrites, which is an indicator of nitrosative stress, increases in many inflammatory disease situations, such as RA (Abdel Jaleel et al., 2021). Serum NO levels were assessed in each of the seven treatment groups at the study endpoint. As demonstrated in Figure 8B, the arthritic control group (immunized with FCA) had a **2.29**-fold greater NO level in the serum compared to the healthy control group. In contrast, the other treatment groups (PEITC NE HG, MTX NP HG, and DD NP HG) had a considerably lower the NO increment. The PEITC NE HG and MTX NP HG treatments significantly reduced the NO level to **1.81**-fold and **1.69**-fold compared to the arthritic control group. While DD NP HG treated group markedly showed a lower level of NO up to **2.12**- fold when compared to the arthritic control group and displayed the highest degree of defense against oxidation (Figure 8B).

Glutathione (GSH, a tripeptide antioxidant enzyme) content in the serum is a valuable indicator for estimating the degree of oxidative stress (Qindeel et al., 2020). Compared to normal control rats, the serum GSH in arthritic control rats was markedly reduced by **3.2-**fold (Figure 8C). Treatment with PEITC NE HG and MTX NP HG demonstrated a considerable rise in serum GSH levels to **2.40-**fold and **2.50**-fold, respectively, compared to the arthritic control rats. The DD NP HG treated rat markedly improved the level of oxidative stress across all treatment groups, with a significant increase in serum GSH up to **2.89**-fold (Figure 8C).

MPO is a known marker for neutrophil infiltration and plays a significant role in the pathophysiology of RA (Aborehab et al., 2017; Li et al., 2019). Compared to the normal healthy control group, the mean serum level of MPO was considerably higher in the arthritic control group (**2.06**-fold). The PEITC NE HG (**1.50**-fold) and MTX NP HG (**1.53**-fold) treatment groups had significantly lower mean serum levels of MPO than the arthritic control group. The highest decline in the mean serum level of MPO was observed in the DD NP HG (**1.94-fold**) (Figure 8D).

According to the results obtained from all the above biochemical parameters, PEITC NE HG and MTX NP HG dramatically reduced oxidative enzymes while improving the level of protective enzymes compared to Free PEITC + Free MTX and BD NP HG. However, DD NP HG enhances the status of oxidative and protective enzymes to a greater extent than the other treatment groups. The localized and site-specific delivery of MTX and PEITC in the nanoparticulate form in inflamed joints may be the basis of DD NP HG’s superior efficacy than Free PEITC and Free MTX.

#### 3.9.3. Effect of dual-drug nanoparticles loaded hydrogel on serum cytokines level in FCA-induced arthritic rats

The underlying etiology of RA is yet unknown. The primary goal of any new treatment approach is to obtain immunological homeostasis in the joint by balancing inflammation and halting the progression of the disease, which has tremendous therapeutic potential. Cytokines and chemokines play essential roles in the advancement and development of RA, whereas suppressing the expression or function of cytokines and chemokine increases the probability of remission of RA and protects bones and cartilage from destruction (Gravallese, 2002; Ren et al., 2021).

**Figure 9.**
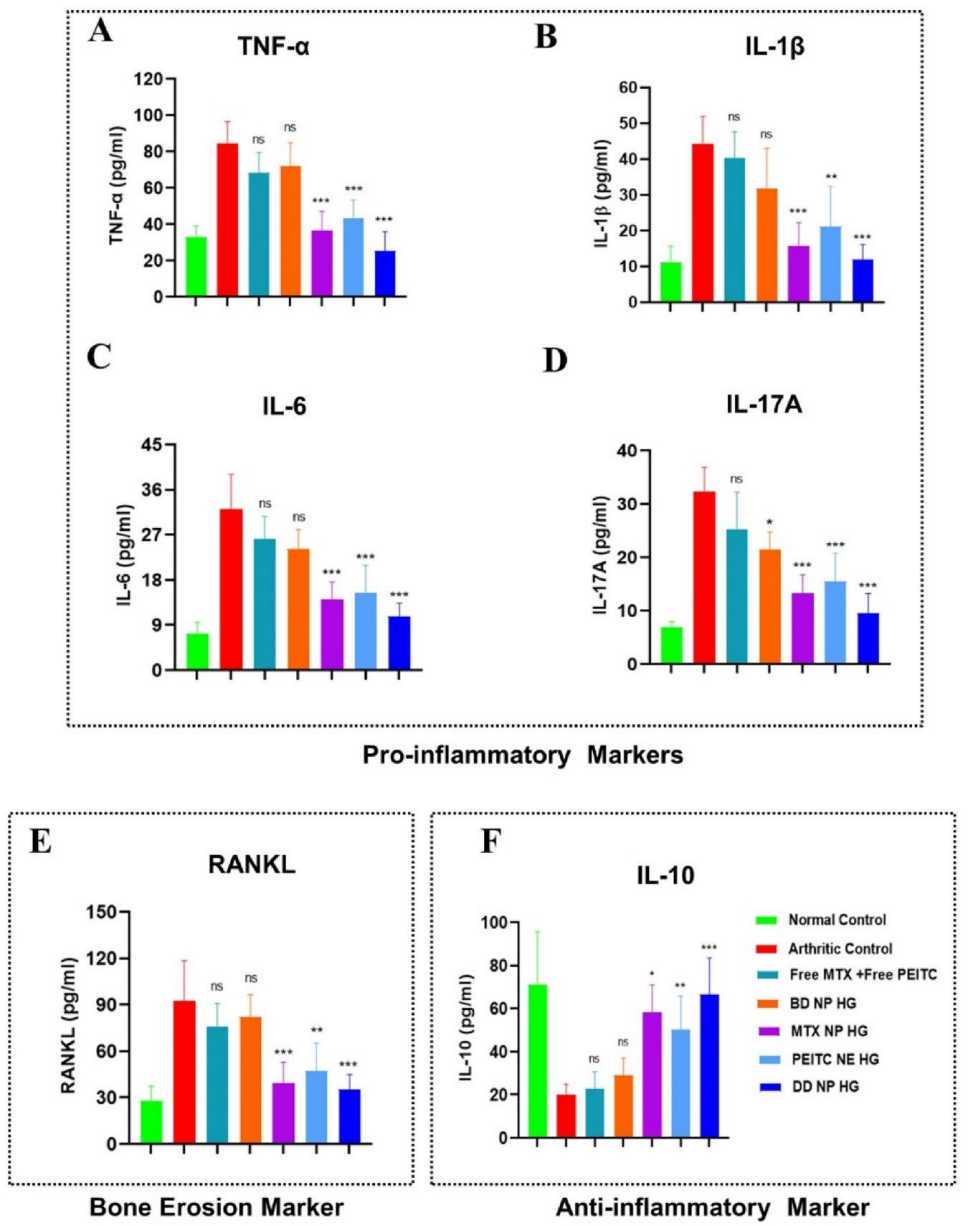
Estimation of pro-inflammatory, anti-inflammatory, and bone erosion markers in FCA-induced RA rats. Effect of dual-drug nanoparticles loaded hydrogel on serum pro-inflammatory cytokine levels (A). TNF-α, (B). IL-1β, (C). IL-6 and (D). IL-17A. (E). RANKL (bone erosion marker) (F). IL-10 (anti-inflammatory cytokine). The serum cytokine level in the rat (on day 33) was estimated by ELISA. The data are represented as mean ± SD (*n* = 4). The values were statistically examined using a one-way ANOVA test. Statistical significance: *p < 0.05, **p < 0.01, ***p < 0.001, and ns- non-significant. TNF-α: Tumor Necrosis Factor-alpha, IL-1β: Interleukin 1 beta, IL-6: Interleukin 6, IL-17A: Interleukin 17A, RANKL: Receptor activator of nuclear factor-kappa-B ligand and IL-10: Interleukin-10, ELISA: Enzyme-linked immunoassay.

In AIA rats, the expression of both pro-inflammatory and anti-inflammatory cytokines was investigated in different treatment groups by ELISA. Here, the serum levels of important pro-inflammatory cytokines, such as TNF-α, IL-6, IL-1β, IL-17A, and IL-10, were determined in the different study groups. In PBS-treated arthritic rats, an increased IL-6, IL-1β, IL-17A, and TNF-α with decreased IL-10 was observed as the disease progressed (Figure 9A and D, F). In comparison to the arthritic control group, no substantial difference was observed although Free PEITC + Free MTX and BD NP HG treatment slightly lowered the level of pro-inflammatory cytokines. Following administration of PEITC NE HG, MTX HG, and DD NP HG, the expression of pro-inflammatory cytokines was markedly reduced, while anti-inflammatory cytokine levels were increased (Figure 9A and D, F).

Our findings also showed that DD NP HG treatment significantly increased the anti-inflammatory cytokine (IL-10) while suppressing the pro-inflammatory cytokines (TNF-α, IL-6, IL-1β, and IL-17A), demonstrating its therapeutic impact was far superior to that of PEITC NE HG or MTX NP HG alone (Figure 9A and D, F).

The RANKL/OPG system plays a pivotal role in joint destruction in RA by regulating the differentiation and function of osteoclasts (Pettit et al., 2006). To elucidate the mechanisms responsible for the joint-protective action, the serum level RANKL was measured to signify the grade of osteoclastogenesis (Stolina et al., 2009). As depicted in Figure 9E, the serum levels of RANKL in AIA model rats (arthritic control) were significantly up-regulated than the healthy control group. Treatments with PEITC NE HG, MTX NP HG, and DD NP HG markedly down-regulated the serum concentration of RANKL. Moreover, RANKL levels in the DD NP HG rats showed the highest decline among the other treatment groups, following the trend of the healthy control group. However, the previous results indicated that DD NP HG could considerably improve the production of cytokines (pro-inflammatory & anti-inflammatory) and bone erosion markers; hence, DD NP HG had the most significant therapeutic impact on preventing the onset of RA.

#### 3.9.4. Toxicity assessment of dual-drug nanoparticles loaded hydrogel in the FCA-induced arthritic rats

**Figure 10.**
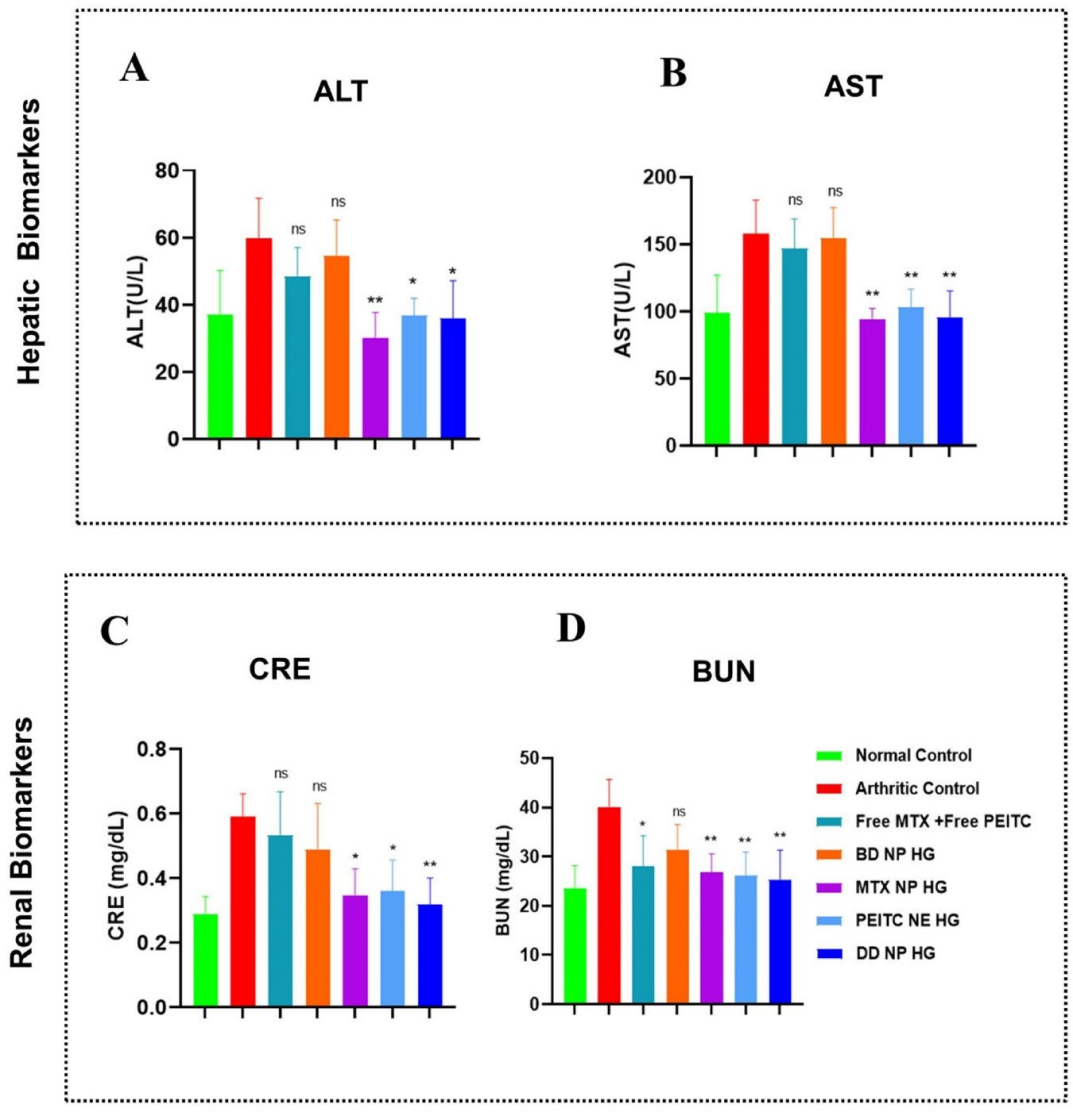
Toxicity assessment in FCA-induced RA rats. Effects of dual-drug nanoparticles loaded hydrogel on hepatic and renal function. (A and B) hepatic biomarkers (ALT and AST) and (C and D) renal biomarkers (CRE and BUN). The data are presented as mean ± SD (*n* = 4). The values were statistically examined using a one-way ANOVA test. Statistical significance: *p < 0.05, **p < 0.01, ***p < 0.001, and ns- non-significant. ALT: Alanine transaminase, AST: Aspartate transaminase, CRE: Creatinine, BUN: Blood urea nitrogen (BUN).

The impact of various treatment groups on liver and kidney functions was evaluated to assess the biocompatibility of nanocarriers for their utilization in biological and medical applications (Yin et al., 2020). As seen in Figures 10A–D, arthritic control rats showed a substantial rise in the liver (ALT and AST) and kidney (CRE and BUN) enzymes level compared to healthy control rats; however, these levels remained within normal ranges. Previously, it was reported that MTX caused liver and kidney function toxicity. Considering the side effects of MTX, we analyzed the impact of MTX-loaded hydrogel treatment groups, i.e. MTX NP HG and DD NP HG, on liver and kidney function. In addition, the blood biochemical analysis demonstrated that the ALT, AST, BUN, and CRE in the groups (MTX NP HG and DD NP HG) were within normal ranges. Hence, we could conclude these hydrogels were effective and safe for *in vivo* RA therapy as they did not affect the liver and kidney functions. By examining the serum biochemical profiles of the other hydrogel treatment groups, BD NP HG and PEITC NE HG, it was determined that all biochemical parameters (ALT, AST, BUN, and CRE) were within the normal ranges, indicating that none of the hydrogel formulations appeared to have a substantially detrimental effect on kidney or liver function (Figure 10A–D). These outcomes demonstrated the safety and biocompatibility of the constructed hydrogel-based carrier system *in vivo*.

#### 3.9.5. Histological analysis of dual-drug nanoparticles loaded hydrogel in the FCA- induced arthritic rats

Rheumatoid arthritis (RA) is the most prevalent form of autoimmune arthritis, characterized by persistent synovitis, cartilage degradation, and bone destruction (Catrina et al., 2016). FCA-induced adjuvant arthritis (AIA) in the rat is a polyarthritis experimental model extensively utilized for preclinical testing of various anti-arthritic drugs that are either being investigated in preclinical or clinical research or are currently being used as treatments in this disease condition (Pearson, 1956). Furthermore, AIA is a type of T-cell-mediated autoimmune arthritis extensively researched for the immunological aspects of RA and other inflammatory or arthritic disorders in humans (van Eden et al., 1996). The local injection of adjuvant (*Mycobacterium tuberculosis* suspended in mineral oil) into rats produces an immune response that typically entails inflammatory degradation of cartilage and bone in the distal joints with concurrent swelling of surrounding tissues.

The animals from the various treatment groups were sacrificed at the end of the study period (i.e. the 33^rd^ day) using CO_2_ asphyxia to verify the therapeutic effectiveness of DD NP HG treatment in FCA-induced arthritic rats. The ankle joints were carefully dissected for gross and histological analysis. The histological assessment of ankles joints was performed with Hematoxylin and Eosin (H&E), Safranin-O/fast green (SO-FG), and Toluidine blue (TB) staining to compare synovial inflammation, cartilage destruction, and bone erosion in all groups microscopically.

**Figure 11.**
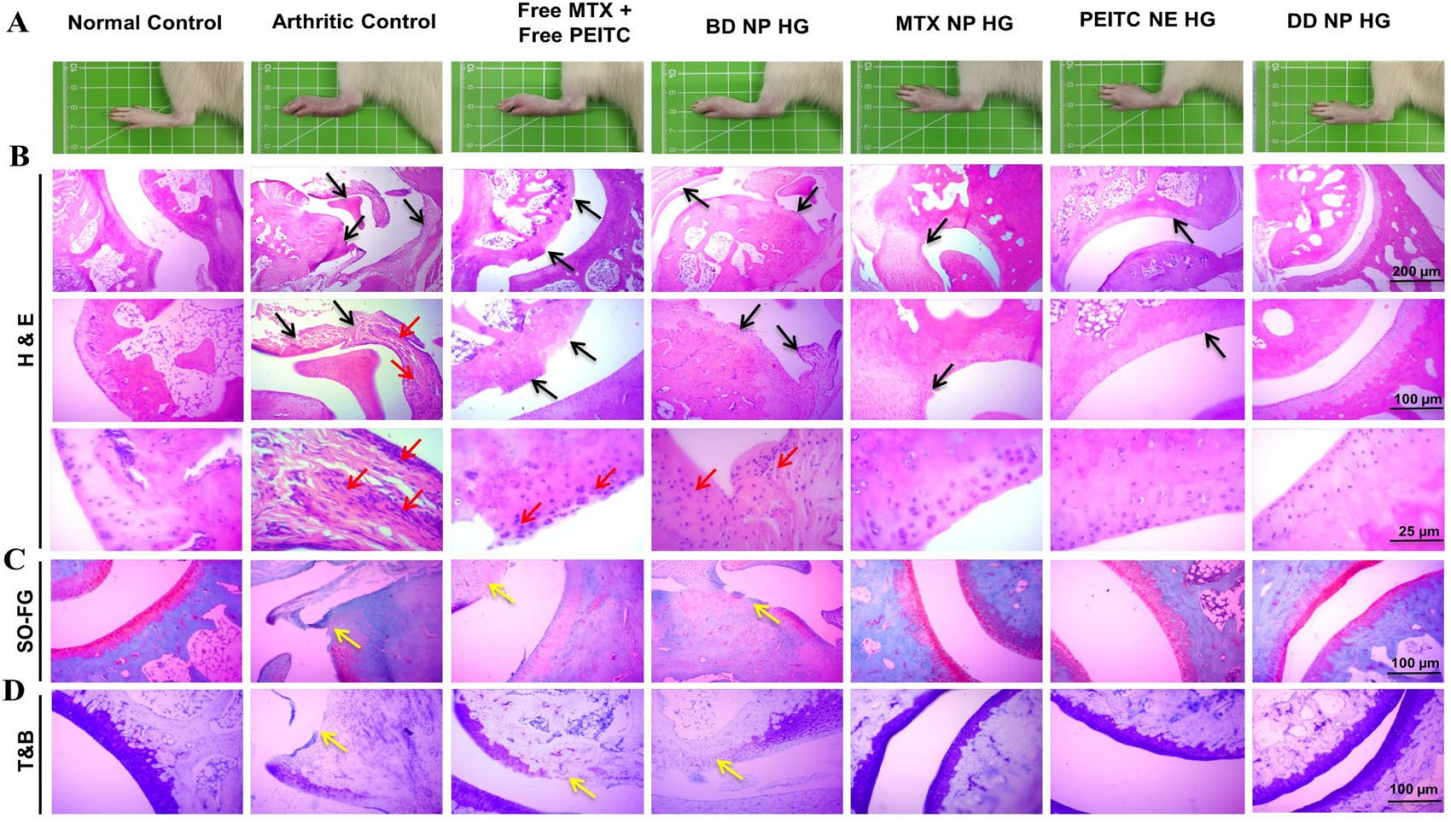
*In vivo* anti-arthritis efficacy of dual-drug nanoparticles loaded hydrogel. Histopathological assessment of bone destruction and cartilage damage in RA model. (A). *In vivo* paw images of the rats from different groups at the endpoint, i.e. 33^rd^ day. Histological sections with (B). H&E, (C). SO-FG and (D). TB staining of ankle joints in different treatment groups. The red arrow indicates inflammatory cellular infiltration, and the black arrow represents bone erosion and cartilage degradation. Yellow arrows indicate loss of proteoglycan. Scale bar = 200 μm, 100 μm and 25 μm. H&E: Hematoxylin and eosin; SO-FG: Safranin O-fast green; T&B: Toluidine blue.

Healthy normal control showed no apparent signs of inflammation, bone erosion, or cartilage destruction. The interface between cartilage and bone in the healthy rats was clearly distinguished by their morphologies (Figure 11B). The arthritic control group had more pronounced severe infiltration of inflammatory cells (red arrows), bone erosion, and cartilage damage (black arrows), which characteristics features of RA (as seen from H&E are staining in Figure 11B) (Zhang et al., 2018). The stained microsections of the ankle joint from the groups treated with the Free MTX+Free PEITC and BD NP HG groups confirmed an intermediary disease state with abundant infiltration of inflammatory cells with considerable bone erosion and cartilage degradation. Treatment with MTX NP HG and PEITC NE HG showed minimal bone erosion and cartilage destruction with nominal cell infiltration in the joint. In addition, the ankle joint sections from DD NP HG-treated rats showed a relatively healthy articular surface with minimal inflammatory cell infiltration. The DD NP HG showed minimal histological alterations compared to other AIA treatment groups and was quite similar to healthy normal control rats without AIA (Figure 11B).

SO-FG is a standard staining method to detect the amount and distribution of proteoglycans in cartilage tissues (Jang et al., 2020). Safranin-O (SO) works by forming a reddish-orange complex when it binds to acidic proteoglycans in the cartilage tissues, such as chondroitin sulfate or keratan sulfate, with a high affinity. A matrix proteoglycan depletion assessment is made possible by the degree of the color of SO, which represents proteoglycan content indirectly (Yin et al., 2020). The joint sections of the PBS-treated rat (arthritic control), Free MTX+Free PEITC, and BD NP HG showed significant proteoglycan loss (the *red* color almost vanished), indicating severe damage and considerable loss of cartilage and bone (Figure 11C). As shown in Figure 11C, minimal cartilage destruction in the rat treated with MTX NP HG and PEITC NE HG, which is evident from the intensity of the *red* color, increased to a different extent. However, the rat treated with DD NP HG had minimal cartilage loss with the highest intensity of *red* color, quite similar to the healthy normal control without AIA induction (Figure 11C).

Another popular method for histologically evaluating differentiated cartilaginous and chondrogenic tissues is TB staining. TB is a cationic dye that visualizes proteoglycans in tissue because its high affinity for the sulfate groups in proteoglycans results in *blue* color (Bergholt et al., 2019). TB staining showed a significant loss of proteoglycan in the arthritic control group, indicating that articular cartilage was severely degraded and damaged (Figure 11D). While in the DD NP HG group, the *blue* color positive areas of TB staining were more prominent compared to Free MTX+Free PEITC and BD NP HG, demonstrating that cartilage injury was effectively reversed, and it could effectively alleviate synovitis and cartilage damage in AIA rats (Figure 11D).

The outcomes from the histopathological studies supported the therapeutic potential of combinational nanocomposite hydrogel (DD NP HG) in reducing inflammation and slowing the onset of RA disease.

## 4. Conclusions

In this study, a dual-nanoparticulate hydrogel formulation of MTX and PEITC (DD NP HG) was successfully prepared and verified for the therapeutic potential of co-delivering of MTX, a slow-acting anti-rheumatic drug, and PEITC, a bioactive phytochemical with potent anti-inflammatory activity, to inflamed joints for synergistic treatment of treating RA. While both drugs are used for treating RA or inflammatory conditions, the expected therapeutic efficacy was never achieved. The *in vitro* data demonstrated that both drugs, in their nanoparticulate form, have the necessary features, such as better mechanical strength, thermosensitivity, injectability, and good biosafety, for prolonged release of MTX and PEITC from the target site, when synthesized as an injectable hydrogel using pluronic and alginate. From the studies conducted on FCA-induced RA in rats, we could demonstrate that the DD NP HG treatment enhanced the efficacy of combination therapy where the inflammatory response was significantly inhibited, reducing chronic inflammation effectively and delaying bone erosion. There are three main advantages of our formulation. First, the current engineered formulation possesses a high therapeutic potential where commercialized pharmaceutical excipients and an inexpensive, simple synthesis approach are applied. Second, the fabrication of the nanoformulation of both MTX and PEITC aided in mitigating the drawbacks and drug-related side effects. Third, as a combination therapy, the simultaneous co-delivery of MTX and PEITC from the hydrogel reduced the dosing intervals and ameliorated the RA disease condition in the inflamed joints of rats.

In conclusion, the result demonstrates that the anti-RA therapy accelerated by the intra-articular injection of DD NP HG can maximize the therapeutic effects and reduce the dose-related side effects of the free form of both the drugs (MTX and PEITC). The possible mechanism can be postulated that the sustained release of nanoparticulate forms of MTX and PEITC from the smart hydrogel resulted in pronounced anti-inflammation over time, eventually leading to amelioration from the RA state.

For the synergistic treatment of rheumatoid arthritis and other metabolic bone degenerative conditions, a combination therapy strategy has a promising future and is urgently needed. This is the first study that reports a combinational nanoparticulate hydrogel formulation for intra-articular injection whose therapeutic efficacy was also characterized and studied in a rodent-RA model.

## Supplementary Material

Supplemental Material
