# Supplementary material for: Formulation of a dual drug-loaded nanoparticulate co-delivery hydrogel system and its validation in rheumatoid arthritis animal model
Source: Drug Deliv. 2023 Feb 28;30(1):2184307. doi: 10.1080/10717544.2023.2184307 (PMC9980407; doi:10.1080/10717544.2023.2184307)
Supplement: Supplemental Material [file IDRD_A_2184307_SM3834.docx]

**Supporting Information**

**Formulation of a dual drug-loaded nanoparticulate co-delivery hydrogel system and its validation in rheumatoid arthritis animal model**

Prakash Haloi ^a, b^, B. Siva Lokesh ^a, b^, Saurabh Chawla ^a, b^, V. Badireenath Konkimalla ^a, b^ *

a School of Biological Sciences, National Institute of Science Education and Research, HBNI, Jatni, Odisha 752050, India

b Homi Bhabha National Institute, Training School Complex, Anushakti Nagar, Mumbai 400094, India

* Corresponding author:

Dr. V. Badireenath Konkimalla

School of Biological Sciences,

National Institute of Science Education & Research (NISER), PO- Bhimpur-Padanpur, Via- Jatni, District:- Khurda, Bhubaneswar, Orissa - 752 050, INDIA

E-mail: [badireenath@niser.ac.in](mailto:badireenath@niser.ac.in) (V.B. Konkimalla)

Tel: +91-674-249 42 11

**2. Materials and methods**

**Materials**

Pluronic F-127 powder (PF-127, Sigma-Aldrich), Sodium alginate (Sigma-Aldrich), EDTA Disodium salt dihydrate, Hi-ARTM (Himedia), Phenethyl isothiocyanate (PEITC, 99 % Sigma-Aldrich), Benzene-1, 2-dithiol (BDT, 96 %), Acetone (EMSURE ACS for analysis, Merck), Ethanol absolute (EMSURE ACS for analysis, Merck), Methanol (EMSURE ACS for analysis, Merck), Formaldehyde, ACS (37 %, MP Biomedicals), Phosphate Buffered Saline (PBS, pH 7.2, liquid sterile filtered, Sigma-Aldrich), Dimethyl sulfoxide (DMSO for HPLC, ≥ 99.7 %, Sigma-Aldrich), Poly-L-lysine solution (0.1 % w/v in water, Sigma-Aldrich), Potassium Bromide (KBr, FTIR Grade, ≥ 99 %, Sigma-Aldrich), Dialysis Membrane -70 (Himedia), Freund′s Adjuvant, Complete (FCA, Sigma-Aldrich), 1,1,3,3-tetramethoxypropane (Sigma-Aldrich), 5,5′-dithiobis(2-nitrobenzoic acid) (DTNB, Sigma-Aldrich), 2-thiobarbituric acid (TBA, Sigma-Aldrich), Methotrexate (MTX, M.P. Biomedicals), Poly-Lacto glycolic acid (PLGA, Sigma-Aldrich), Olive oil (Himedia), polysorbate-80 (Himedia), Span-80 (Himedia) and D-Mannitol(Merck).

**Abbreviations**

BDT- 1, 2 Benzenedithiol; DMSO- Dimethyl sulfoxide; HLB - Hydrophilic–lipophilic balance; BL HG - Blank hydrogel; BD NP HG – Blank dual nanoparticles loaded hydrogel; DD NP HG- Dual-drug nanoparticles loaded hydrogel; HG- Hydrogel; MTX- Methotrexate; NE – Nanoemulsion; NP- Nanoparticle; PEITC- Phenethyl isothiocyanate; PLGA- Poly-Lacto glycolic acid; PS-Particle size; PDI- Polydispersity index; ZP- Zeta potential; SA- Sodium alginate

**Methods**

*2.1. Fabrication of blank and MTX-loaded PLGA nanoparticles (MTX NP)*

The nanoprecipitation technique was applied to fabricate Methotrexate (MTX)-loaded PLGA nanoparticles (NP). In brief, different amounts (80-150 mg) of PLGA (shown in Table 1) were dissolved in a solvent mixture of acetone and DMSO (9:1), followed by the addition of MTX, and vortexed until a clear solution was obtained. The above organic phase was added drop-by-drop into an aqueous phase (20 mL) containing 0.1 % w/v PF-127 surfactant under continuous stirring (at 800 rpm) until complete removal of organic solvent. The obtained nanoparticles were dialyzed against 10 % DMSO to remove unloaded Free MTX, followed by dialysis against water to remove small residuals of DMSO. Finally, the obtained MTX-loaded PLGA nanoparticles (**MTX NP**) were lyophilized using Mannitol (cryoprotectant) and stored at 4 °C.

*2.2. Preparation of PEITC o/w nanoemulsion (PEITC NE) using different surfactant concentrations*

A nanoemulsion of PEITC was prepared and optimized by varying the concentration of surfactants (Span-80: Tween-80) shown in Table S1**.** Different surfactant mixtures were selected based on the hydrophilic–lipophilic balance (HLB) values between 4 to 15 and further used for nanoemulsion preparation. The procedure involved specific amounts of olive oil and surfactant mixture taken in a glass vial and subjected to stirring for 1 h at 1200 rpm. Subsequently, under constant stirring, the aqueous phase was added dropwise to the oil phase, and stirring was continued for another 3 h. The obtained blank nanoemulsion was probe sonicated at 60 % amplitude (with a pulse of 5 s ON and 5s OFF) for 30 min [1].

Further optimized blank nanoemulsion was used to prepare the PEITC nanoemulsion (**PEITC NE**), in which PEITC was added in the oil phase along with olive oil. A similar procedure was employed as that of blank nanoemulsion.

*2.3. Characterization of drug-loaded nanosystems*

*2.3.1. Determination of entrapment efficiency (%) and drug loading (%) of MTX-PLGA nanoparticles (MTX NP)*

The prepared drug-loaded nanoparticles were placed in a dialysis bag (MWCO 10 kDa) and dialyzed against 10 % DMSO in a static condition for 4 h. Finally, the concentration of Free MTX was determined using a UV-visible spectrophotometer (BioSpectrometer® kinetic Eppendorf, Hamburg, Germany) at 302 nm [2].

*2.3.2. Drug content for the optimized PEITC nanoemulsion (PEITC NE)*

The PEITC content in the nanoemulsion was determined by taking 10 µL of PEITC NE, diluting it to 100 µL with DMSO, and vortexed thoroughly until a clear solution. The obtained clear solution containing PEITC was analyzed using a UV-visible spectrophotometer (BioSpectrometer® kinetic Eppendorf, Hamburg, Germany) using BDT assay at 365 nm [3].

*2.3.3. BDT assay*

Briefly, 30 µL of the sample was added with 125 µL of potassium phosphate buffer and 125 µL of BDT in methanol. Later, the samples were vortexed for 1 min and heated in a dry bath at 65 °C for 2 h. Upon completion of the reaction, the samples were centrifuged at 10,000 rpm and analyzed using a UV-vis spectrophotometer at 365 nm [2].

2.3.4. *Particle size, PDI, and Zeta potential*

Particle size (PS), polydispersity index (PDI), and zeta potential (ZP) were determined by preparing different dilutions of samples using Milli Q water at a room temperature of 25 °C with a backscattered angle of 173° using Zetasizer Nano ZS (Malvern Instruments, Malvern, UK) [4].

*2.4. Fabrication of dual-drug nanoparticles loaded hydrogel*

Dual-drug nanoparticles loaded hydrogel (**DD NP HG**) was prepared according to the method previously described by Yin *et al*. 2020a [5]. Briefly, blank PLGA NP was uniformly dispersed in the o/w nanoemulsion by sonication for 10-15 min. After the dispersion, 1 % SA was added and mixed uniformly at 600 rpm until homogenous dispersion. To the above nanoparticular SA dispersion, different concentrations of PF-127 (shown in Table 3) were added in cold conditions (4-10 °C) and stirred continuously at 1000 rpm for 24 h to obtain the blank dual nanoparticles loaded hydrogel (**BD NP HG**). A similar procedure was followed to fabricate MTX-PLGA nanoparticle loaded hydrogel (**MTX NP HG**), PEITC nanoemulsion loaded hydrogel (**PEITC NE HG**), and **DD NP HG**. The different hydrogels obtained were stored at a refrigerated temperature (4 °C) [5–7].

*2.5. Characterization of dual-drug nanoparticles loaded hydrogel*

*2.5.1. Fourier Transform Infrared Spectroscopy (FTIR)*

FTIR studies for free Methotrexate (Free MTX), free Phenethyl isothiocyanate (Free PEITC), BD NP HG, MTX NP HG, PEITC NE HG, and DD NP HG were analyzed to investigate the incorporation of drug or chemical interactions between the drug and excipients using FT-IR spectrophotometer (Nicolet iS5, Thermo Scientific, USA) in which each sample was mixed with KBr and compressed into a pellet using a die. Each sample pellet was further scanned individually at an average of 16 scans at a resolution of 4 cm^-1^ over a wavelength of 4000-500 cm^-1^. The spectra were processed and analyzed using Origin software (Origin 8.1).

*2.5.2. Determination of drug content*

The amounts of drug loaded into the different hydrogels were estimated using the centrifugation method. Briefly, 100 µL of the gel was taken, and the contents were dissolved entirely in 1 mL DMSO. Later, it was centrifuged at 10,000 rpm for 15 min, and the obtained clear supernatant was subjected to different dilutions using DMSO and measured by a UV-visible spectrophotometer. MTX was determined at 302 nm, and PEITC was analyzed using BDT assay at 365 nm, as described previously in section 2.3.3. [3].

*2.5.3. Solubility studies*

The aqueous solubility studies were carried out between the free MTX, free PEITC, and the optimized dual-drug nanoparticles loaded hydrogel (DD NP HG) using the shaking method. The specified amount of hydrogel was taken, equivalent to both the unprocessed free drugs, and then dispersed in the water. Further, the shaking was done at 100 rpm for 1 h, and the dispersions were centrifuged at 10,000 rpm for 15 min. Finally, the amount of drug present in the supernatant was examined using a UV-visible spectrophotometer at 302 nm for MTX and 365 nm for BDT-conjugated PEITC [3]. (BDT assay as discussed earlier in section no: 2.3.3)

*2.5.4. Determination of gelation time*

The time taken for the transition of sol-phase hydrogel (flowing fluid) to gel-phase (non-flowing gel) is known as gelation time. It was determined by the tube inversion method in a water bath at a constant temperature of 25 °C and 37 °C (Julabo ED, Germany). In brief, a glass vial containing 2 mL hydrogel of each sample was dipped in a water bath, and fluidity was checked by tilting the tube every 15 s. The time the hydrogel (solution form) was phase transitioned to gel form at both temperatures is recorded and expressed as mean ± SD (n=3) [8].

*2.5.5. In vitro* degradation studies

The *in vitro* degradation profile of different hydrogel formulations was performed by observing the behavior of gels in their wet form using the shaking method. Initially, 1 mL of hydrogel formulation (in sol form) was taken in a 5 mL glass vial and left undisturbed for 30 min at 37 °C to form a gel. About 3 mL of PBS (0.1 M, pH 7.4) was added to the gels and gently stirred (50 rpm, 37 °C) in a shaking incubator (New Brunswick™ Excella® E24 Shaker Series, Eppendorf AG, Germany). After every 24 h, the buffer solution was removed to weigh the hydrogels, followed by a fresh addition of PBS (3 mL) to the individual vials. A similar procedure was continued until > 80 % degradation of the hydrogel. The procedure was continued every 24 h for 6 days. The degradation of the following hydrogels was estimated by using equation 1

(1)

%Biodegradation = $\frac{(W_{i}-W_{d})}{W_{d}}\times100$

*W_i_* represents the initial weight of the hydrogel, and *W_d_* represents the weight of the degraded hydrogel

All the experiments were performed in triplicates, and the results were presented as mean ± SD. A graph was plotted between the % weight of the gel remaining versus time in days [9,10].

*2.5.6. Injectability of hydrogels*

The injectability of BD NP HG, MTX NP HG, PEITC NE HG, and DD NP HG was evaluated by observing thread-like hydrogel formation on injection of the respective hydrogel into a vial containing PBS (pH 7.2-7.4) at 37 °C using a syringe (24-gauge needle) [11].

*2.5.7. Morphological characterization of the dual-drug nanoparticles loaded hydrogel*

The morphology of the blank hydrogel (BL HG) and DD NP HG was analyzed using field emission scanning electron microscopy (FESEM). The samples were frozen at −80 °C for 12 h and kept in a freeze-dryer for 36 h (FreeZone 4.5, Labconco, USA) [12–14]. The dried materials were uniformly spread on a silicon wafer and coated with gold under a vacuum. The gold-coated silicon wafer was then pasted on a metallic stub for analysis using a FESEM (ZEISS GeminiSEM 450, Carl Zeiss, Germany). The analysis was performed at an operating voltage of 3.5 kV under a high vacuum.

*2.5.8. Rheological properties of the dual-drug nanoparticles loaded hydrogel*

The rheological properties of the hydrogels were performed using a HR-30 rheometer (TA instruments). The storage modulus (G′) and loss modulus (G′′) were recorded during a temperature ramp from 25 °C to 45 °C, with a rate of 3 °C/min. Additionally, the complex viscosity (η*) measurements were performed at 25 ℃ and 37 ℃, with a range of angular frequencies (ω: 0.1-100 rad/s) for the injectable application. For G′ and G′′ of frequency dependencies, the data were collected for the modulus with a frequency range of a range of angular frequencies (ω: 0.1-100 rad/s) at 37 °C [15,16].

*2.5.9. In-vitro drug release and kinetics studies*

The release profiles of MTX and PEITC from different formulations were evaluated *in vitro* at pH 7.4 in PBS with 0.5 % Tween-80 [5]. Briefly, 250 µL of the different samples (MTX NP HG, PEITC NE HG, and DD NP HG) containing 1 mg MTX and 10 mg PEITC were placed into a dialysis membrane (MWCO 10 kDa) and allowed to form a gel (at 37 °C). The dialysis bag with the sample gel was suspended in a beaker containing 50 mL dissolution medium (PBS with 0.5 % tween-80) and placed in a shaker incubator with constant shaking at 100 rpm maintained at physiological body conditions (37 °C, pH-7.4). Samples were withdrawn at different time intervals 0, 1, 2, 4, 6, 8, 12, 24, 36, 48, 72, 96, and 120 h. The specific amount of drug released at different time points was analyzed using a UV-visible spectrophotometer at 302 nm for MTX and 365 nm for BDT-conjugated PEITC (BDT assay, described in section no: 2.3.3). To determine the drug release kinetics from the hydrogels, the results were then plotted in a variety of kinetic models, including Zero-order, first-order, Higuchi, Hixon-Crowell, and Korsmeyer-Peppas models.

*2.5.10. Stability studies*

The physical stability of the drug NPs loaded hydrogels (MTX NP HG, PEITC NE HG, and DD NP HG) was investigated by storing the nanoparticular hydrogels at 4 °C for 60 days. The samples were withdrawn at predetermined intervals and estimated for percentage drug loading (% DC). The stability of the hydrogel was assessed by studying the variations in the final drug content [17].

*2.6. Animals*

Healthy SD rats (180-235 g b.w.) were used for all the *in vivo* experiments. The animals were housed in pathogen-free controlled climatic conditions with artificial lighting (12:12 h of light: dark cycle). The rats were individually kept and acclimatized for 7 days before the experiment. Clean drinking water and a commercially available standard pellet diet were provided *ad libitum*. All animal experiments were carried out in compliance with ARRIVE guidelines and were performed per the National Institutes of Health (NIH) guide for the care and use of Laboratory animals (NIH Publications No. 8023, revised 1978). The experimental protocols were performed in accordance with CPCSEA regulations (Registration No. 1634/GO/ReBi/S/12/CPCSEA, DoR- 29.03.2012) and were approved by the Institutional Animal Ethics Committee (IAEC) at the National Institute of Science Education and Research (NISER), Bhubaneswar, Odisha, India (Ethical Approval No: NISER/SBS/AH-222).

2.7. *In vivo therapeutic efficacy of dual-drug nanoparticles loaded hydrogel*

*2.7.1. Adjuvant-Induced Arthritis (AIA) induction and monitoring*

The adjuvant-Induced Arthritis (AIA) rat model was established by injecting Freund's Complete Adjuvant (FCA) in female SD (180-235 g b.w.) rats. Briefly, a 250 µL of FCA solution has injected subcutaneously into the sub-plantar region of the left hind paw under deep isoflurane anesthesia except for the rats serving as normal control (administered 250 µL of sterile PBS). The animals were regularly monitored, and the arthritis parameters were measured from day 4 to day 33 following FCA immunization [18–22].

After 12 days of the arthritis induction, the rats with uniform swelling and redness in the FCA-injected paws were selected for the study [20,22]. The rats were randomly divided into seven groups (n = 4), (i) normal healthy control group (normal control), (ii) arthritis group (FCA-challenged and injected with sterile PBS, arthritis control, (iii) PEITC-nanoemulsion loaded hydrogel treated group (PEITC NE HG) challenged with FCA, (iv) MTX nanoparticle loaded hydrogel treated group (MTX NP HG) challenged with FCA, (v) Free PEITC and Free MTX (Free PEITC + Free MTX) treated group challenged with FCA, (vi) dual-drug nanoparticles loaded hydrogel treated group challenged with FCA (DD NP HG), and (vii) blank dual nanoparticles loaded hydrogel (BD NP HG) treated group challenged with FCA. The treatments were started from day 12 by intra-articularly (IA) injecting 50 µL of the test substances directly into the knee joint area of arthritic rats on days 12, 17, 22, and 27 after FCA immunization [23].

*2.7.2. Assessment of arthritis progression upon FCA administration*

The paw edema in each rat was examined at specific time points on the 12^th^, 15^th^, 20^th^, 25^th^, 30^th,^ and 33^rd^ day after administration of different formulations [24,25]. Paw edema volume (expressed in *ml)* was measured by making a visible mark on each paw at the tibiotarsal joint and dipping the paw at the marked level. The displaced volumes were recorded using a digital plethysmometer (WBP01, Vihan Techno Services, India). The paw thickness (expressed in *mm)* was measured using a digital micrometer (Mitutoyo, Japan) at defined time points. The body weight of rats was measured using a standard digital weighing balance. Every four days, from day 0 to day 32, the body weight of each animal was recorded and shown as (*gms*) [26].

The arthritis score was calculated by microscopically observing the degree of swelling and redness of joint edema of periarticular tissues in the FCA-injected and non-injected hind paws. The arthritis score was assigned based on the following observations: a). '0' for normal with no erythema and swelling; b). '1' for swollen digits with erythema; c). '2' for mild swelling of limbs and erythema, d). '3' for moderate swelling observed from the ankle extending to the metatarsophalangeal or metacarpophalangeal joints, and e). '4' for severe swelling observed extending from the ankle to the digits, culminating in ankylosis and loss of joint movement. The arthritis score for an individual rat was calculated as the sum of the four paw scores (a maximum score taken as 16 per rat). The severity of arthritis and therapeutic efficacy of the formulation was assessed based on the scores obtained by the rats from the treatment groups (on day 12, 15, 20, 25, 30 and 33) [27–29].

*2.7.3. Blood collection and serum separation*

Blood was collected from each rat by retro-orbital plexus bleeding under isoflurane anesthesia and sacrificed by CO_2_ asphyxiation on day 33. The blood samples were then transferred in a sterile 1.5 mL tube and centrifuged at 3000 rpm for 10 min at 4 °C (Centrifuge 5424 R, Eppendorf AG, Germany). The collected serum samples assessed different biochemical parameters and indicators for oxidative stress and inflammation[30–32].

*2.7.4. Determination of thymus and spleen index*

The rats from the various treatment groups were sacrificed using CO_2_ asphyxia on the final day (33^rd^ day) to isolate the immune organs (thymus and spleen). The organs were weighed to determine the thymus or spleen index, which is calculated as follows: [31,33,34].

(2)

Spleen index (%) = $\frac{Spleen weight}{Body weight} \times100$

(3)

Thymus index (%) = $\frac{Thymus weight}{Body weight} \times100$

*2.7.5. Evaluation of oxidative stress*

Oxidative stress in the animals was assessed by estimating the serum levels of different biochemical markers such as malondialdehyde (MDA), nitric oxide (NO), glutathione (GSH), and myeloperoxidase (MPO). MDA is one of the thiobarbituric acid-reactive substances (TBARS) generally used for estimating lipid peroxidation. Lipid peroxidation in the serum was determined by following a standard protocol where the formation of malondialdehyde-thiobarbituric acid (MDA-TBA) adduct upon reacting MDA with TBA in acidic conditions at 100 °C. The absorbance of the reaction end product (TBARS) was determined at 532 nm using a spectrophotometer. 1,1,3,3-tetra methoxy propane was used as the standard, with the results expressed as µM of TBARS [21,35–37]. Serum Nitric oxide (NO) was measured by Griess assay using a commercial Nitrite/Nitrate Assay Kit (Sigma-Aldrich, USA) and performed as per the manufacturer's instructions [21,38,39]. Estimation of glutathione (GSH) in the serum was performed by the standard dithionitrobenzoic acid (DTNB) method [40,41]. Here, the sulfhydryl groups in the samples, when mixed with DTNB, formed a yellow color solution detected at 412 nm. The concentration of GSH (in µM) was calculated from the standard GSH curve. Myeloperoxidase (MPO) activity in serum was detected following the protocol provided by the manufacturer (Elabscience, MD, USA).

*2.7.6. Evaluation of cytokines levels in the serum*

The relative serum levels of representative pro-inflammatory cytokines, anti-inflammatory cytokines, and bone erosion markers were performed using an ELISA [21,28,42,43]. Here, interleukins (IL-1β, IL-17A, IL-6 IL-10) and receptor activator of NF-κB ligand (RANKL) were measured using commercially available rat-specific ELISA kits from Elabscience, MD, USA, and Invitrogen, Thermo Fisher Scientific, MI, USA in case of tumor necrosis factor-α (TNF-α) as per manufacturer's instruction.

The cytokines were detected using serum (50 µL) from the animals in the various treatment groups. The procedures and data analyses were followed as per the manufacturer. A multimode microplate spectrophotometer (Thermo Scientific, Varioskan Flash, USA) was used to measure absorbance at 450 nm, and cytokine concentrations were estimated using a standard curve.

*2.7.7. Biochemical test to assess liver and kidney function*

Following a 33 day study period, the serum levels of different biochemical markers were used to determine hepatotoxicity and nephrotoxicity in different treatment animal groups. Hepatotoxicity, i.e., alanine aminotransferase (ALT), aspartate aminotransferase (AST), and nephrotoxicity, i.e., blood urea nitrogen (BUN), and creatinine (CRE) were measured in the serum of RA animals using commercially available kits according to the manufacturer's instructions (Meril Diagnostics). These biochemical parameters were tested using a Merilyzer Cliniquant, a semi-automated biochemical analyzer [32,34].

*2.7.8. Histopathological assessment*

At the end of the study, the rats were sacrificed to dissect the hind limbs of each group. The excised hind limbs were preserved in 10 % neutral buffered formalin and later decalcified with 14 % neutral EDTA solution. The decalcified tissue embedded in paraffin blocks was sliced using an automated Rotary Microtome (HistoCore AUTOCUT, Leica Biosystems, Germany). On the sliced sections Hematoxylin and eosin (H&E), Safranin O-fast green (SO-FG), and Toluidine blue (T&B) staining were performed according to standard protocols for microscopical observations [18,44].

*2.8. Statistical analysis*

All quantitative parameters are presented as the mean ± SD. GraphPad Prism 8.0 (GraphPad Software, La Jolla, CA, USA) was used for statistical analysis. Comparative analysis between multiple groups was carried out by one-way analysis of variance (ANOVA) followed by Dunnett's multiple comparisons test. A significant difference is considered when the p-value is less than 0.05 (*p < 0.05).

**References**

[1] Y. Li, Z. Teng, P. Chen, Y. Song, Y. Luo, Q. Wang, Enhancement of aqueous stability of allyl isothiocyanate using nanoemulsions prepared by an emulsion inversion point method, J. Colloid Interface Sci. 438 (2015) 130–137. https://doi.org/10.1016/j.jcis.2014.09.055.

[2] S. Mohanty, A.K. Sahoo, V.B. Konkimalla, A. Pal, S.C. Si, Naringin in combination with isothiocyanates as liposomal formulations potentiates the anti-inflammatory activity in different acute and chronic animal models of rheumatoid arthritis, ACS Omega. 5 (2020) 28319–28332. https://doi.org/10.1021/acsomega.0c04300.

[3] U.F. Aly, H.A. Abou-Taleb, Ah.A.H. Abdellatif, N.S. Tolba, Formulation and evaluation of simvastatin polymeric nanoparticles loaded in hydrogel for optimum wound healing purpose, Drug Des. Devel. Ther. 13 (2019) 1567–1580. https://doi.org/10.2147/DDDT.S198413.

[4] F. Danhier, P. Danhier, N. Schleich, C. Po, S. Laurent, P. Sibret, C. Jérôme, V. Poucelle, B. Gallez, V. Préat, Tumor Targeting by RGD-Grafted PLGA-Based Nanotheranostics Loaded with Paclitaxel and Superparamagnetic Iron Oxides, in: Methods Pharmacol. Toxicol., 2018. https://doi.org/10.1007/7653_2015_43.

[5] N. Yin, X. Guo, R. Sun, H. Liu, L. Tang, J. Gou, T. Yin, H. He, Y. Zhang, X. Tang, Intra-articular injection of indomethacin-methotrexate: In situ hydrogel for the synergistic treatment of rheumatoid arthritis, J. Mater. Chem. B. 8 (2020) 993–1007. https://doi.org/10.1039/c9tb01795j.

[6] M. Qindeel, N. Ahmed, F. Sabir, S. Khan, A. Ur-Rehman, Development of novel pH-sensitive nanoparticles loaded hydrogel for transdermal drug delivery, Drug Dev. Ind. Pharm. 45 (2019) 629–641. https://doi.org/10.1080/03639045.2019.1569031.

[7] S. Vignesh, A. Sivashanmugam, M. Annapoorna, R. Janarthanan, I. Subramania, N. Shantikumar V., R. Jayakumar, Injectable deferoxamine nanoparticles loaded chitosan-hyaluronic acid coacervate hydrogel for therapeutic angiogenesis, Colloids Surfaces B Biointerfaces. 161 (2018) 129–138. https://doi.org/10.1016/j.colsurfb.2017.10.033.

[8] H. Qin, J. Wang, T. Wang, X. Gao, Q. Wan, X. Pei, Preparation and characterization of chitosan/β-glycerophosphate thermal-sensitive hydrogel reinforced by graphene oxide, Front. Chem. 6 (2018) 565. https://doi.org/10.3389/fchem.2018.00565.

[9] Y. Mao, X. Li, G. Chen, S. Wang, Thermosensitive Hydrogel System with Paclitaxel Liposomes Used in Localized Drug Delivery System for in Situ Treatment of Tumor: Better Antitumor Efficacy and Lower Toxicity, J. Pharm. Sci. 105 (2016) 194–204. https://doi.org/10.1002/jps.24693.

[10] Y. Qu, J. Tang, L. Liu, L.L. Song, S. Chen, Y. Gao, α-Tocopherol liposome loaded chitosan hydrogel to suppress oxidative stress injury in cardiomyocytes, Int. J. Biol. Macromol. 125 (2019) 1192–1202. https://doi.org/10.1016/j.ijbiomac.2018.09.092.

[11] W. Chen, K. Shi, J. Liu, P. Yang, R. Han, M. Pan, L. Yuan, C. Fang, Y. Yu, Z. Qian, Sustained co-delivery of 5-fluorouracil and cis-platinum via biodegradable thermo-sensitive hydrogel for intraoperative synergistic combination chemotherapy of gastric cancer, Bioact. Mater. 23 (2023) 1–15. https://doi.org/10.1016/j.bioactmat.2022.10.004.

[12] S. Yu, X. Zhang, G. Tan, L. Tian, D. Liu, Y. Liu, X. Yang, W. Pan, A novel pH-induced thermosensitive hydrogel composed of carboxymethyl chitosan and poloxamer cross-linked by glutaraldehyde for ophthalmic drug delivery, Carbohydr. Polym. 155 (2017) 208–217. https://doi.org/10.1016/j.carbpol.2016.08.073.

[13] C. Qian, T. Zhang, J. Gravesande, C. Baysah, X. Song, J. Xing, Injectable and self-healing polysaccharide-based hydrogel for pH-responsive drug release, Int. J. Biol. Macromol. 123 (2019) 140–148. https://doi.org/10.1016/j.ijbiomac.2018.11.048.

[14] P. Pankongadisak, O. Suwantong, Enhanced properties of injectable chitosan-based thermogelling hydrogels by silk fibroin and longan seed extract for bone tissue engineering, Int. J. Biol. Macromol. 138 (2019) 412–424. https://doi.org/10.1016/j.ijbiomac.2019.07.100.

[15] X. Ke, M. Li, X. Wang, J. Liang, X. Wang, S. Wu, M. Long, C. Hu, An injectable chitosan/dextran/β -glycerophosphate hydrogel as cell delivery carrier for therapy of myocardial infarction, Carbohydr. Polym. 229 (2020) 115516. https://doi.org/10.1016/j.carbpol.2019.115516.

[16] M.H. Monteiro do Nascimento, F.N. Ambrosio, D.C. Ferraraz, H. Windisch-Neto, S.M. Querobino, M. Nascimento-Sales, C. Alberto-Silva, M.A. Christoffolete, M.K.K.D. Franco, B. Kent, F. Yokaichiya, C.B. Lombello, D.R. de Araujo, Sulforaphane-loaded hyaluronic acid-poloxamer hybrid hydrogel enhances cartilage protection in osteoarthritis models, Mater. Sci. Eng. C. Mater. Biol. Appl. 128 (2021) 112345. https://doi.org/10.1016/j.msec.2021.112345.

[17] P. Mura, N. Mennini, C. Nativi, B. Richichi, In situ mucoadhesive-thermosensitive liposomal gel as a novel vehicle for nasal extended delivery of opiorphin, Eur. J. Pharm. Biopharm. 122 (2018) 54–61. https://doi.org/10.1016/j.ejpb.2017.10.008.

[18] C. Deng, Q. Zhang, P. He, B. Zhou, K. He, X. Sun, G. Lei, T. Gong, Z. Zhang, Targeted apoptosis of macrophages and osteoclasts in arthritic joints is effective against advanced inflammatory arthritis, Nat. Commun. 12 (2021) 2174. https://doi.org/10.1038/s41467-021-22454-z.

[19] S.M.A. El-Sheikh, A.E.A.F. Abd El-Alim, A.A.A. Galal, R.G. El-Sayed, N.I. El-naseery, Anti-arthritic effect of β-caryophyllene and its ameliorative role on methotrexate and/or leflunomide-induced side effects in arthritic rats, Life Sci. 233 (2019) 116750. https://doi.org/10.1016/j.lfs.2019.116750.

[20] A. Zeb, O.S. Qureshi, C.H. Yu, M. Akram, H.S. Kim, M.S. Kim, J.H. Kang, A. Majid, S.Y. Chang, O.N. Bae, J.K. Kim, Enhanced anti-rheumatic activity of methotrexate-entrapped ultradeformable liposomal gel in adjuvant-induced arthritis rat model, Int. J. Pharm. 525 (2017) 92–100. https://doi.org/10.1016/j.ijpharm.2017.04.032.

[21] H.S. Helmy, A.E. El-Sahar, R.H. Sayed, R.N. Shamma, A.H. Salama, E.M. Elbaz, Therapeutic effects of lornoxicam-loaded nanomicellar formula in experimental models of rheumatoid arthritis, Int. J. Nanomedicine. 12 (2017) 7015–7023. https://doi.org/10.2147/IJN.S147738.

[22] S. Pandey, V. Kumar, A. Leekha, N. Rai, F.J. Ahmad, A.K. Verma, Co-Delivery of Teriflunomide and Methotrexate from Hydroxyapatite Nanoparticles for the Treatment of Rheumatoid Arthritis: In Vitro Characterization, Pharmacodynamic and Biochemical Investigations, Pharm. Res. 35 (2018) 201. doi: 10.1007/s11095-018-2478-2.

[23] J. Kim, H.Y. Kim, S.Y. Song, S.H. Go, H.S. Sohn, S. Baik, M. Soh, K. Kim, D. Kim, H.C. Kim, N. Lee, B.S. Kim, T. Hyeon, Synergistic Oxygen Generation and Reactive Oxygen Species Scavenging by Manganese Ferrite/Ceria Co-decorated Nanoparticles for Rheumatoid Arthritis Treatment, ACS Nano. 13 (2019) 3206–3217. https://doi.org/10.1021/acsnano.8b08785.

[24] F. Vincenzi, M. Padovan, M. Targa, C. Corciulo, S. Giacuzzo, S. Merighi, S. Gessi, M. Govoni, P.A. Borea, K. Varani, A2A Adenosine Receptors Are Differentially Modulated by Pharmacological Treatments in Rheumatoid Arthritis Patients and Their Stimulation Ameliorates Adjuvant-Induced Arthritis in Rats, PLoS One. 8 (2013) e54195. https://doi.org/10.1371/journal.pone.0054195.

[25] K.J. Gou, R. Zeng, Y. Dong, Q.Q. Hu, H.W.Y. Hu, K.G. Maffucci, Q.L. Dou, Q.B. Yang, X.H. Qin, Y. Qu, Anti-inflammatory and analgesic effects of Polygonum orientale L. extracts, Front. Pharmacol. 8 (2017) 562. https://doi.org/10.3389/fphar.2017.00562.

[26] T. Pan, T. fang Cheng, Y. ran Jia, P. Li, F. Li, Anti-rheumatoid arthritis effects of traditional Chinese herb couple in adjuvant-induced arthritis in rats, J. Ethnopharmacol. 205 (2017) 1–7. https://doi.org/10.1016/j.jep.2017.04.020.

[27] J. Asenso, J. Yu, F. Xiao, M. Zhao, J. Wang, Y. Wu, C. Wang, W. Wei, Methotrexate improves the anti-arthritic effects of Paeoniflorin-6’-O-benzene sulfonate by enhancing its pharmacokinetic properties in adjuvant-induced arthritis rats, Biomed. Pharmacother. 112 (2019) 108644. https://doi.org/10.1016/j.biopha.2019.108644.

[28] Z.F. Wei, X.L. Jiao, T. Wang, Q. Lu, Y.F. Xia, Z.T. Wang, Q.L. Guo, G.X. Chou, Y. Dai, Norisoboldine alleviates joint destruction in rats with adjuvant-induced arthritis by reducing RANKL, IL-6, PGE 2, and MMP-13 expression, Acta Pharmacol. Sin. 34 (2013) 403–413. https://doi.org/10.1038/aps.2012.187.

[29] C. Qian, M. Kuang, Y. Wang, Effect of Qianghuo Erhuang Decoction on T Regulatory and T Helper 17 Cells in Treatment of Adjuvant-induced Arthritis in Rats, Sci. Rep. 7 (2017) 17198. https://doi.org/10.1038/s41598-017-17566-w.

[30] U.H. Hassan, Alamgeer, M. Shahzad, A. Shabbir, S. Jahan, M. Saleem, I.A. Bukhari, A.M. Assiri, Amelioration of adjuvant induced arthritis in Sprague Dawley rats through modulation of inflammatory mediators by Ribes alpestre Decne, J. Ethnopharmacol. 235 (2019) 460–471. https://doi.org/10.1016/j.jep.2019.02.025.

[31] L. Yang, R. Liu, A. Fan, J. Zhao, Y. Zhang, J. He, Chemical Composition of Pterospermum heterophyllum Root and its Anti-Arthritis Effect on Adjuvant-Induced Arthritis in Rats via Modulation of Inflammatory Responses, Front. Pharmacol. 11 (2020) 584849. https://doi.org/10.3389/fphar.2020.584849.

[32] N. Yin, X. Tan, H. Liu, F. He, N. Ding, J. Gou, T. Yin, H. He, Y. Zhang, X. Tang, A novel indomethacin/methotrexate/MMP-9 siRNA: In situ hydrogel with dual effects of anti-inflammatory activity and reversal of cartilage disruption for the synergistic treatment of rheumatoid arthritis, Nanoscale. 12 (2020) 8546–8562. https://doi.org/10.1039/d0nr00454e.

[33] R. Jing, Y. Ban, W. Xu, H. Nian, Y. Guo, Y. Geng, Y. Zang, C. Zheng, Therapeutic effects of the total lignans from Vitex negundo seeds on collagen-induced arthritis in rats, Phytomedicine. 58 (2019) 152825. https://doi.org/10.1016/j.phymed.2019.152825.

[34] Y. Wang, Z. Liu, T. Li, L. Chen, J. Lyu, C. Li, Y. Lin, N. Hao, M. Zhou, Z. Zhong, Enhanced therapeutic effect of RGD-modified polymeric micelles loaded with low-dose methotrexate and nimesulide on rheumatoid arthritis, Theranostics. 9 (2019) 708–720. https://doi.org/10.7150/thno.30418.

[35] K. Yagi, Simple assay for the level of total lipid peroxides in serum or plasma., Methods Mol. Biol. 108 (1998) 101–106. https://doi.org/10.1385/0-89603-472-0:101.

[36] M. Uchiyama, M. Mihara, Determination of malonaldehyde precursor in tissues by thiobarbituric acid test, Anal. Biochem. 86 (1978) 271–278. https://doi.org/10.1016/0003-2697(78)90342-1.

[37] M.K. Mazumder, R. Paul, P. Bhattacharya, A. Borah, Neurological sequel of chronic kidney disease: From diminished Acetylcholinesterase activity to mitochondrial dysfunctions, oxidative stress and inflammation in mice brain, Sci. Rep. 9 (2019) 3097. https://doi.org/10.1038/s41598-018-37935-3.

[38] A. Brizzolari, M. Dei Cas, D. Cialoni, A. Marroni, C. Morano, M. Samaja, R. Paroni, F.M. Rubino, High-Throughput Griess Assay of Nitrite and Nitrate in Plasma and Red Blood Cells for Human Physiology Studies under Extreme Conditions., Molecules. 26 (2021) 4569. https://doi.org/10.3390/molecules26154569.

[39] K.M. Miranda, M.G. Espey, D.A. Wink, A rapid, simple spectrophotometric method for simultaneous detection of nitrate and nitrite, Nitric Oxide - Biol. Chem. 5 (2001) 62–71. https://doi.org/10.1006/niox.2000.0319.

[40] G.L. Ellman, Tissue sulfhydryl groups, Arch. Biochem. Biophys. 82 (1959) 70–77. https://doi.org/10.1016/0003-9861(59)90090-6.

[41] M.S. Moron, J.W. Depierre, B. Mannervik, Levels of glutathione, glutathione reductase and glutathione S-transferase activities in rat lung and liver, BBA - Gen. Subj. 582 (1979) 67–78. https://doi.org/10.1016/0304-4165(79)90289-7.

[42] J. Yeo, Y.M. Lee, J. Lee, D. Park, K. Kim, J. Kim, J. Park, W.J. Kim, Nitric Oxide-Scavenging Nanogel for Treating Rheumatoid Arthritis, Nano Lett. 19 (2019) 6716–6724. https://doi.org/10.1021/acs.nanolett.9b00496.

[43] Q. Linghang, X. Yiyi, C. Guosheng, X. Kang, T. Jiyuan, L. Xiong, W. Guangzhong, L. Shuiqing, L. Yanju, Effects of Atractylodes Oil on Inflammatory Response and Serum Metabolites in Adjuvant Arthritis Rats, Biomed. Pharmacother. 127 (2020) 110130. https://doi.org/10.1016/j.biopha.2020.110130.

[44] S. Ren, H. Liu, X. Wang, J. Bi, S. Lu, C. Zhu, H. Li, W. Kong, R. Chen, Z. Chen, Acupoint nanocomposite hydrogel for simulation of acupuncture and targeted delivery of triptolide against rheumatoid arthritis, J. Nanobiotechnology. 19 (2021) 409. https://doi.org/10.1186/s12951-021-01157-z.

**Supporting Figures and Tables**

| 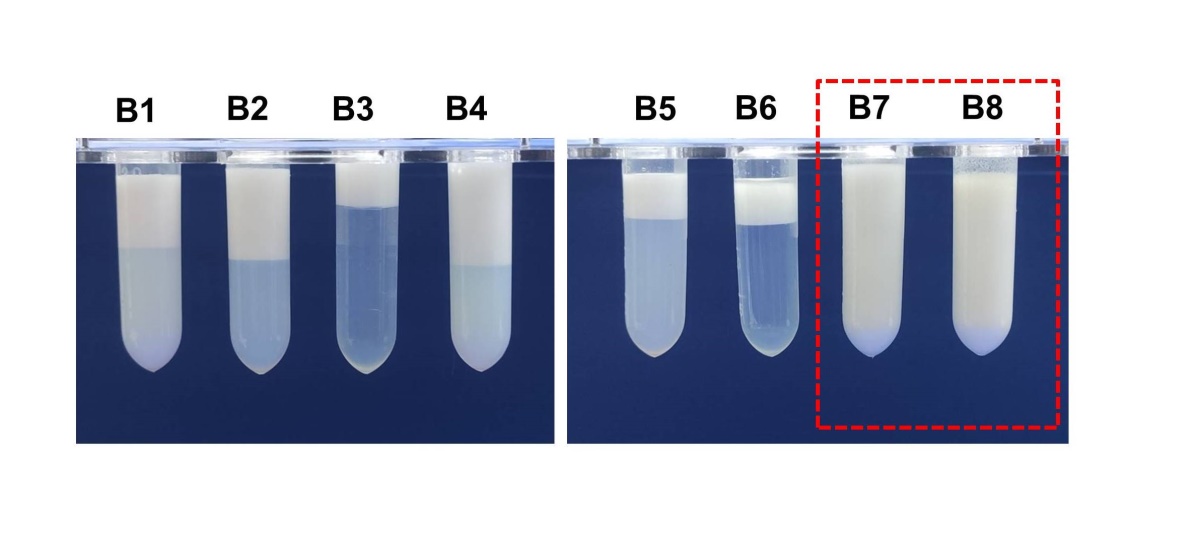 |
| --- |
| **Figure S1.** Observation of creaming and phase separation in the preparation of different PEITC nanoemulsion (PEITC NE) batches |

**Table S1.** Different surfactant concentrations for the preparation of nanoemulsion

| **Batch no** | **Constants** | **Variables** | | **Creaming/ phase separation** | **Particle size (PS)**  **(nm)** |
| --- | --- | --- | --- | --- | --- |
|  |  | **Surfactant 1**  **(% Span 80)** | **Surfactant 2**  **(% Tween 80)** |  |  |
| **1** | Aqueous phase-72%  Oil phase- 18% | 6 | 4 | Observed | Not determined |
| **2** |  | 8 | 2 | Observed | Not determined |
| **3** |  | 2 | 8 | Observed | Not determined |
| **4** |  | 9 | 1 | Observed | Not determined |
| **5** |  | 3 | 7 | Observed | Not determined |
| **6** |  | 1 | 9 | Observed | Not determined |
| **7** |  | 9.7 | 0.3 | Not observed | 303.5 ± 5.12 |
| **8** |  | 10 | 0 | Not observed | 240.67 ± 4.75 |
| **Batch-8 (optimized PEITC NE)** - Drug content = 83.16 ± 10.87 % (33.4 ± 4.35 mg). | | | | | |

| 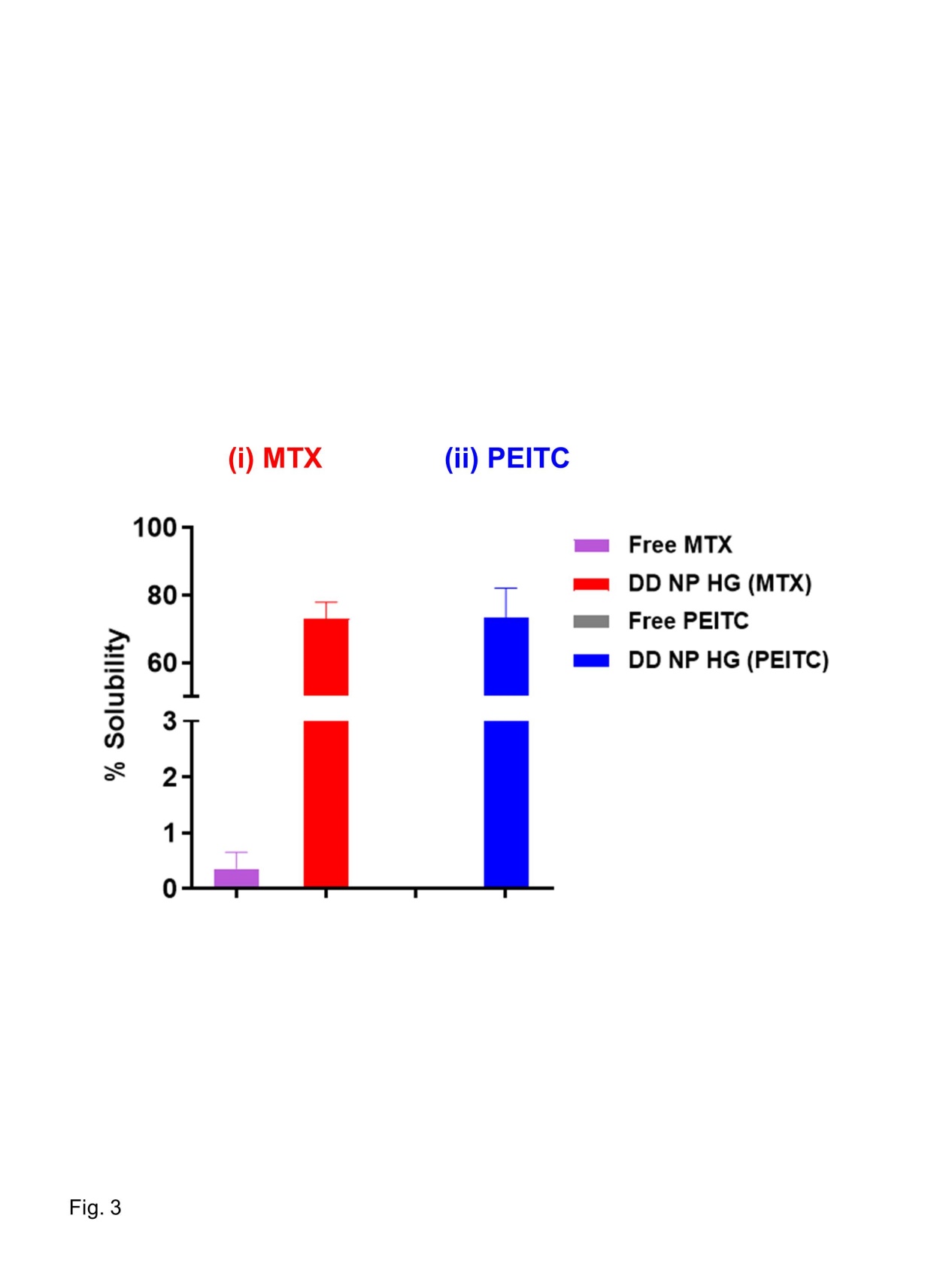 |
| --- |
| **Figure S2.** **Percentage (%) Solubility studies** of **MTX** from (i). Free MTX of DD NP HG and **PEITC** from (ii). Free PEITC of DD NP HG was performed, and respective amounts were calculated spectrophotometrically. All the data are represented as mean ± SD (n = 3). |

| *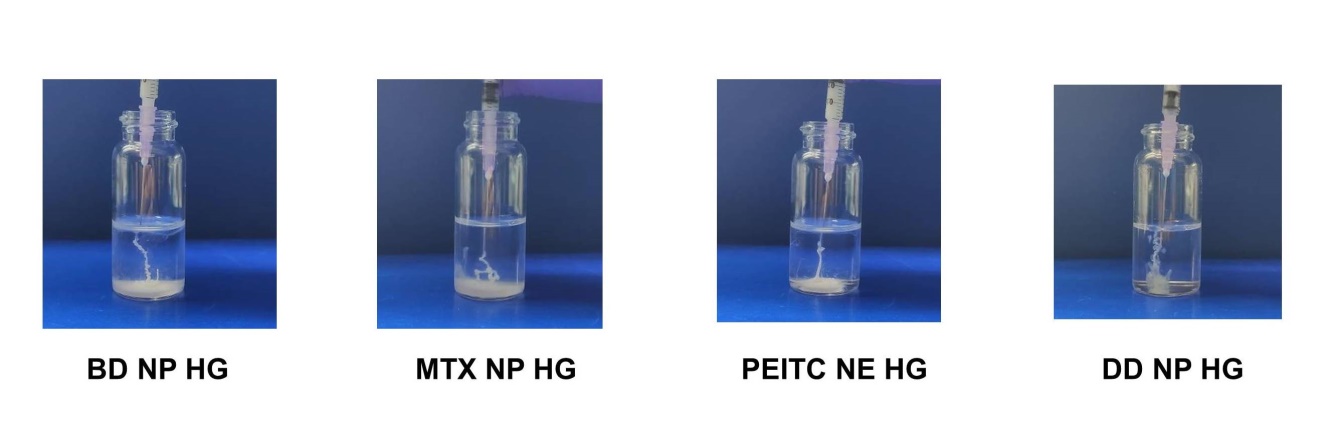* |
| --- |
| **Figure S3. Injectable performance** of the different hydrogel formulations studied at body physiological conditions (pH- 7.4 and 37 °C). |

| 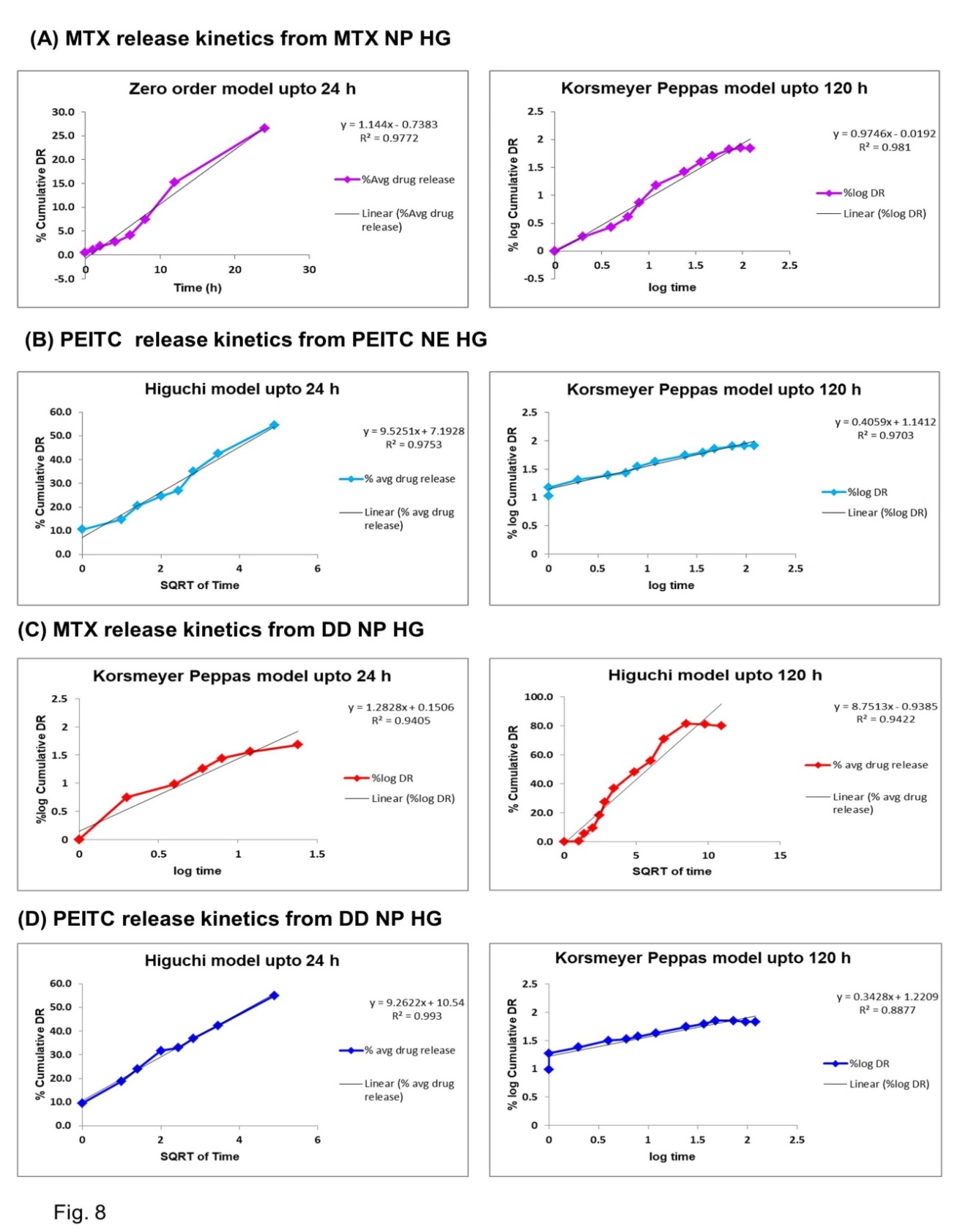 |
| --- |
| **Figure S4.** **The drug release kinetics of dual-drug nanoparticles loaded hydrogel *in vitro****.* Percentage release kinetics (%) MTX and PEITC release from the hydrogel was calculated using different kinetics models at (A) MTX release kinetics (%) from MTX NP HG at 24 h, and 120 h (B) PEITC release kinetics (%) from PEITC NE HG at 24 h and 120 h (C) MTX release kinetics (%) from DD NP HG at 24 h and 120 h and (D) PEITC release kinetics (%) from DD NP HG at 24 h and 120 h. *Magenta color:* MTX release rate from MTX NP HG, *Light blue color:* PEITC release rate from PEITC NE HG, *Red color:* MTX release rate from DD NP HG, and *Navy blue color:* PEITC release rate from DD NP HG. All the data are represented as mean ± SD (n = 3). |

**Table S2.** Release kinetic models and their R^2^ values of different hydrogel formulations

***- indicates the particular formulation following that release model or kinetics

| **Name of the model** | **R^2^ values of MTX from DD NP HG** | | **R^2^ values of PEITC from DD NP HG** | | **R^2^ values of MTX from MTX NP HG** | | **R^2^ values of PEITC from PEITC NE HG** | |
| --- | --- | --- | --- | --- | --- | --- | --- | --- |
|  | **24 h** | **120 h** | **24 h** | **120 h** | **24 h** | **120 h** | **24 h** | **120 h** |
| **Zero-order** | 0.909 | 0.796 | 0.863 | 0.671 | 0.977^***^ | 0.833 | 0.929 | 0.804 |
| **First order** | 0.621 | 0.465 | 0.633 | 0.505 | 0.868 | 0.616 | 0.778 | 0.636 |
| **Higuchi** | 0.934 | 0.942^***^ | 0.993^***^ | 0.877 | 0.837 | 0.941 | 0.975^***^ | 0.953 |
| **Hixon-Crowell** | 0.936 | 0.864 | 0.909 | 0.710 | 0.974 | 0.935 | 0.957 | 0.875 |
| **Korsmeyer-Peppas** | 0.940^***^ | 0.891 | 0.853 | 0.887^***^ | 0.968 | 0.981^***^ | 0.955 | 0.970^***^ |

**Table S3.** Stability studies at 4 °C for different formulations for 60 days. Data are represented as mean ± SD (n=3).

| **Formulation** | **Time (days)** | **DC (%)** | | **Amount of drug present in the hydrogel (mg)** | |
| --- | --- | --- | --- | --- | --- |
| **MTX NP HG** | 0 | 90.9 ± 3.8 | | 3.63 ± 0.07 | |
|  | 30 | 86.7 ± 2.7 | | 3.47 ± 0.11 | |
|  | 60 | 82.2 ± 4.6 | | 3.29 ± 0.18 | |
| **PEITC NE HG** | 0 | 85.7 ± 3.7 | | 34.25 ± 1.35 | |
|  | 30 | 78.3 ± 4.0 | | 31.32 ± 1.59 | |
|  | 60 | 75.6 ± 4.3 | | 30.21 ± 1.72 | |
|  |  | **PEITC** | **MTX** | **PEITC** | **MTX** |
| **DD NP HG** | 0 | 81.8 ± 7.2 | 88.3 ± 9.3 | 32.7 ± 1.4 | 3.53 ± 0.37 |
|  | 30 | 75.7 ± 4.0 | 86.6 ± 9.0 | 30.26 ± 1.6 | 3.46 ± 0.36 |
|  | 60 | 71.7 ± 5.8 | 83.7 ± 5.8 | 28.7 ± 2.32 | 3.35 ± 0.23 |
